# Expanding the Efficacy of Fingermark Enhancement Using ToF-SIMS

**DOI:** 10.3390/molecules28155687

**Published:** 2023-07-27

**Authors:** Deborah Charlton, Catia Costa, Steven J. Hinder, John F. Watts, Melanie J. Bailey

**Affiliations:** 1 Department of Chemistry, University of Surrey, Guildford GU2 7XH, Surrey, UK; d.charlton@surrey.ac.uk; 2 Fingerprint Development Laboratory, Thames Valley Police, Kidlington OX5 2NX, Oxfordshire, UK; 3 Surrey Ion Beam Centre, University of Surrey, Guildford GU2 7XH, Surrey, UK; 4 School of Mechanical Engineering Sciences, University of Surrey, Guildford GU2 7XH, Surrey, UK

**Keywords:** fingerprint, fingermark, secondary ion mass spectrometry, SIMS

## Abstract

Time-of-flight secondary ion mass spectrometry (ToF-SIMS) has been shown to enhance fingermark recovery compared to standard processes used by police forces, but there is no data to show how generally applicable the improvement is. Additionally, ToF-SIMS can be run in either positive or negative ion mode (or both), and there is no data on which mode of operation is most effective at revealing fingerprints. This study aims to fill these gaps by using ToF-SIMS to image fingerprints deposited on two common exhibit-type surfaces (polyethylene and stainless steel) using 10 donors and ageing fingerprints in either ambient, rainwater, or underground for 1 and 5 months. In all, 120 fingerprints were imaged using ToF-SIMS, and each was run in positive and negative modes. A fingerprint expert compared the fingerprint ridge detail produced by the standard process to the ToF-SIMS images. In over 50% of the samples, ToF-SIMS was shown to improve fingerprint ridge detail visualised by the respective standard process for all surfaces tested. In over 90% of the samples, the ridge detail produced by ToF-SIMS was equivalent to standard development across all different ageing and exposure conditions. The data shows that there is a benefit to running the ToF-SIMS in both positive and negative modes, even if no ridge detail was seen in one mode.

## 1. Introduction

A fingermark is a residue left behind by an unknown donor’s finger on a surface, such as a crime scene exhibit. The quality of fingermarks varies, and latent fingermarks are often not visible without enhancement. The visibility of fingermarks depends on factors such as the surface they were deposited on, the initial composition of the residue, and subsequent exposure conditions [1]. The fingermark residue varies among donors and consists of sweat, sebaceous components, and external contaminants [2].

Stainless steel and plastic are common surfaces encountered in forensic laboratories, and the Fingermark Visualisation Manual (FVM) recommends two processes for these surfaces: cyanoacrylate (CA) fuming followed by Basic Yellow 40 (BY40) staining or black powder suspension (BPS). If the surface is wet, black powder suspension is recommended instead of CA fuming. However, the success of visualisation techniques such as cyanoacrylate fuming and black powder suspension is limited by their interaction with specific constituents in the residue. Therefore, only partial development may occur, or be completely absent, if the abundance of these constituents is not high enough or homogenous across the fingermark [3,4,5,6].

Despite numerous physical and chemical techniques used for developing fingermarks, the quality of development of many fingermarks is insufficient for their capture and comparison at the fingerprint bureau. Mass spectrometry imaging techniques, including time-of-flight secondary ion mass spectrometry (ToF-SIMS) and matrix-assisted laser desorption ionisation mass spectrometry imaging (MALDI-MSI), allow molecules in the uppermost surface layers of a sample to be mapped based on their mass to charge ratio (*m*/*z*). The distribution of molecules across the surface can yield an image of fingerprint ridge detail where the composition differs between the residue and the background surface [7,8,9,10].

Category A techniques recommended by the FVM have undergone extensive evaluation and are effective, while lower categories require further evaluation. ToF-SIMS is currently listed as a Category C process in the FVM, which means that it shows potential for effective fingermark recovery [2,3]. In contrast, MALDI-MSI is a Category B process in the FVM [11]. ToF-SIMS can complement MALDI-MSI in several ways. ToF-SIMS is suited to imaging inorganic species (e.g., potassium and chlorine, common endogenous fingerprint residue constituents which are unsuitable for analysis by MALDI-MSI). In addition, ToF-SIMS has superior spatial resolution compared to MALDI-MSI and, therefore, may yield more defined ridge detail. Finally, ToF-SIMS causes minimal destruction to the fingerprint and does not require a matrix to be applied prior to analysis [12]. This could be an advantage for valuable or sentimental exhibits that must be preserved and may also provide some advantages in terms of the preservation of other traces.

The application of ToF-SIMS to fingermarks has been demonstrated previously by Szynkowska et al., Lee et al., and Bailey et al. [13,14,15]. Whilst previous work has demonstrated the potential of ToF-SIMS to provide higher sensitivity compared to Category A processes [16,17], there is no data to show how generally applicable this improvement is. The chemical composition of fingerprints collected from crime scenes is known to vary as a function of the donor, time since deposition (age), environmental exposure, and deposition substrate, for example. These factors are known to affect the efficacy of any subsequent fingerprint development process and, therefore, should be explored to guide forensic practitioners [18,19,20,21,22]. 

The goal of this study is, therefore, to determine how ToF-SIMS imaging could be incorporated into existing Category A workflows, considering the exhibit’s condition. This study aims to expand on previous work on ToF-SIMS by evaluating its effectiveness in sequence with selected Category A processes on multiple donors, increased ageing time points, and environmental exposure conditions. Specifically, this study examines fingerprints from ten donors on two evidentially representative substrate types aged for 1 and 5 months in water, underground burial, and ambient conditions [16]. The scale of this study is unprecedented for ToF-SIMS imaging of fingerprints, and 120 samples have been imaged in both positive and negative modes, with the aim of informing forensic practitioners on the circumstances under which ToF-SIMS can enhance fingerprint visualisation.

## 2. Results and Discussion

### 2.1. Sequential Enhancement by ToF-SIMS Following Standard Processes

In police laboratories, while treatments can be used individually to maximise evidence recovery, techniques can also be performed in sequence, whereby a second development process is applied to a fingermark that is not recovered successfully using a first technique. Due to the high cost of ToF-SIMS instrumentation and lack of availability in police labs, we anticipate that in operational use, ToF-SIMS will always be used after failure to develop a fingermark using a standard process. The technique would be most efficiently employed in workflows where it can be targeted at a partially visualised fingerprint. Each ToF-SIMS image reported in this work took 36 min 48 s to generate and so it would likely be employed after other quicker and cheaper standard processes. 

To assess the added benefit of ToF-SIMS used sequentially after Category A techniques, one fingerprint from each depletion series was selected for ToF-SIMS analysis. The fingerprints selected for ToF-SIMS imaging were those for which the standard process had visualised the least ridge detail. In cases where no development was visualised by the standard process, the first deposition was selected for ToF-SIMS imaging. ToF-SIMS imaging was carried out after CA fuming and BY40 staining for both stainless steel and polyethylene substrates aged in ambient conditions. For those aged in water and soil, ToF-SIMS imaging was carried out after treatment with BPS. The images of all 120 selected samples after standard processes and ToF-SIMS are in Supporting Information Table S1.

To generate the best images with ToF-SIMS, a combination of 10 or more ions was sometimes used, but on other occasions, a good image could be obtained using a single ion, as shown in Table 1. The images shown in Table 1 also provide the opportunity to compare stainless steel and polyethylene substrates as the fingerprints are from the same donor, ageing time, and environmental conditions. Interestingly, the development by CA and BY40 is better on stainless steel, whereas the SIMS imaging performs best on polypropylene. One possible explanation for this is that the poor CA development on the polypropylene leaves the endogenous components of the fingerprint exposed and accessible to the ToF-SIMS. In contrast, the well-developed fingerprint provides poorer quality SIMS images because the CA covers the fingerprint and is not as readily detectable by SIMS.

Through comparison of the images, each fingerprint was assigned a “ToF-SIMS enhancement score”: fingerprints showing no enhancement scored 0, and fingerprints for which ToF-SIMS provided an enhancement scored 1. Some examples of enhanced fingerprints are shown in Table 2.

**Figure 1:**
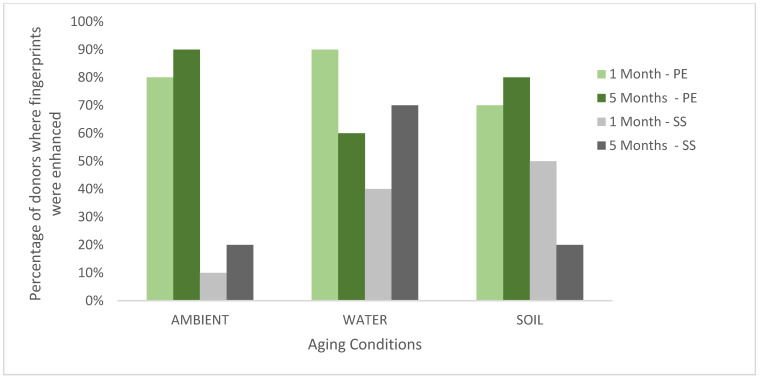
The percentage of donors (n = 10) where the fingerprint was enhanced in the ToF-SIMS imaging for stainless steel (SS) and polyethylene (PE) aged in ambient conditions, water and soil for 1 month or 5 months.

**Figure 2:**
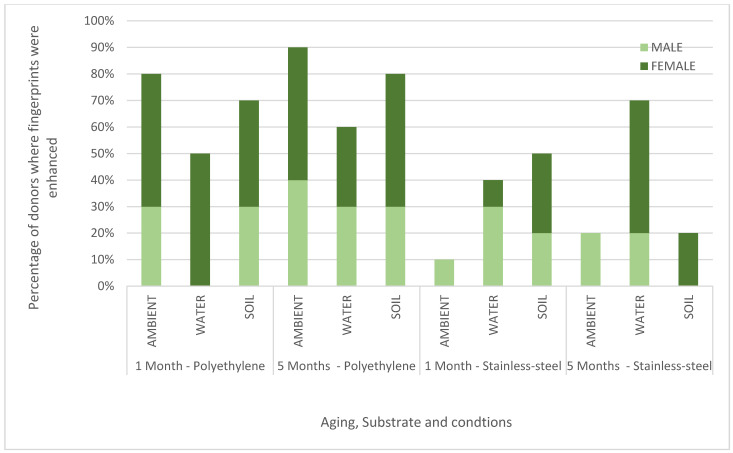
Chart showing the percentage of male and female donors (n = 10) where the fingerprint was enhanced in the ToF-SIMS imaging for stainless steel (SS) and polyethylene (PE) aged in ambient conditions, water and soil for 1 month or 5 months.

The enhancement scores for each substrate, environmental condition, and ageing point are summarised for each donor in Figure 1 which shows the percentage of donors for which ToF-SIMS provided an enhancement. These results are also plotted graphically with females denoted F; males denoted M in Figure 2.

Across all donors, substrates, and conditions (a total of 120 samples), >90% of ToF-SIMS images were determined to be the same or better than those provided by the standard process. In 56% of the samples, the ridge detail was enhanced by ToF-SIMS, providing a strong indication that ToF-SIMS can benefit current category A workflows. The results in Figure 1 show that, in general, the enhancement rate for ToF-SIMS is better for fingerprints deposited on polyethylene (an insulating material) than stainless steel (a conductive material). This is somewhat surprising as ToF-SIMS imaging generally performs better on conducting surfaces, as shown in previous work [16]. It is possible that the standard processes perform better on stainless steel, and so there is less room for improvement, or it may be that the components of the fingerprint residue that ToF-SIMS is particularly sensitive to persist better on polyethylene after treatment. Figure 1 also shows that ToF-SIMS enhanced fingerprints equally well after 1 and 5 months of ageing. Figure 2 shows that overall, ToF-SIMS enhanced fingerprints equally well for both male and female donors used in this study, although no enhancement was seen with female donor fingerprints on stainless steel aged in ambient conditions. 

Overall, more enhancement by ToF-SIMS was seen after ageing in water. As enhancement could be seen in all three conditions and both substrates, this would suggest ToF-SIMS could be incorporated into multiple workflow charts within the FVM for nonporous substrates. Surfaces would have to be smooth and flat to avoid affecting the geometry (i.e., the distance from sample to detector causing flight time to vary).

**Figure 3:**
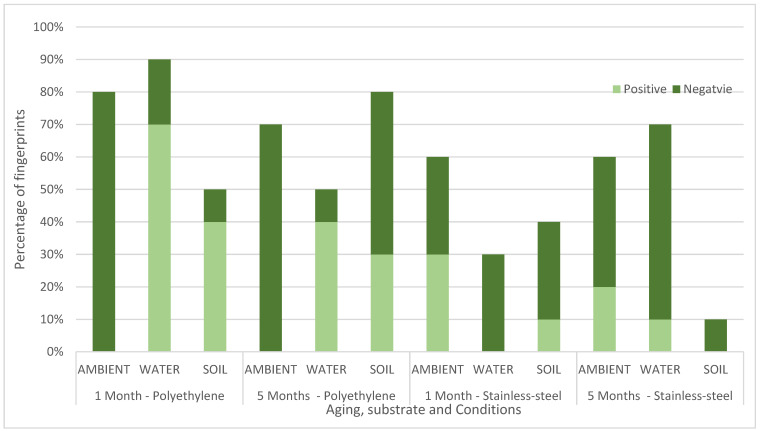
Chart comparing the percentage of fingerprints (n = 10) where ridge was seen better using negative mode or positive mode in the ToF-SIMS imaging for stainless steel (SS) and polyethylene (PE) aged in ambient conditions, water and soil for 1 month or 5 months.

ToF-SIMS can be run in positive or negative mode. Running in both modes doubles the analysis time, and so Figure 3 shows which mode (positive or negative) gave the best images, broken down by substrate, ageing time and environmental condition. For the fingerprints deposited on polyethylene and aged in ambient, negative mode gave better images consistently. This suggests that in operational work for fingerprints on polyethylene thought to have been exposed only to ambient conditions, successful enhancement of fingerprints could be achieved by using negative mode only. However, for other conditions, the results were more mixed, suggesting that both modes should be used to maximise recovery. Overall negative mode gave the best images for 22 male and 22 female fingerprints, compared to 10 and 14, respectively, for positive mode.

For the ambient aged samples, CA was used as an enhancement process prior to SIMS imaging. Some *m*/*z* values (e.g., 177, 224 and 235, assigned to C_9_H_9_O_2_N_2_, C_11_H_14_O_4_N, and C_11_H_11_O_4_N_2_, respectively), used to make up several of the negative ion images treated with CA, can be attributed to fragments associated with cyanoacrylate [23]. In some images, the peaks at *m*/*z* 39 (assigned to ^39^K), *m*/*z* 23 (assigned to ^23^Na), and *m*/*z* 35 (assigned to ^35^Cl), presumed to derive from the salts commonly found in eccrine secretions [2], contribute to the ion image and may have been preserved from washing by the sebaceous components of fingerprint residue or the cyanoacrylate. *m*/*z* 369 contributed to several ion images for female donors and is presumed to be cholesterol [12]. 

Supporting Information Tables S2 and S3 show a table of “common ions” detected in positive and negative mode, respectively, broken down by donor gender, substrate, and ageing condition. The ion was deemed to be “common” if present in five or more samples. A higher frequency of common ions was detected in the one-month-aged samples than in the five-month-aged samples, showing that some species may be lost with increased ageing. These common ions were more prevalent in the ambient aged samples than in the water and soil, which could suggest they are water-soluble. 

**Figure 4:**
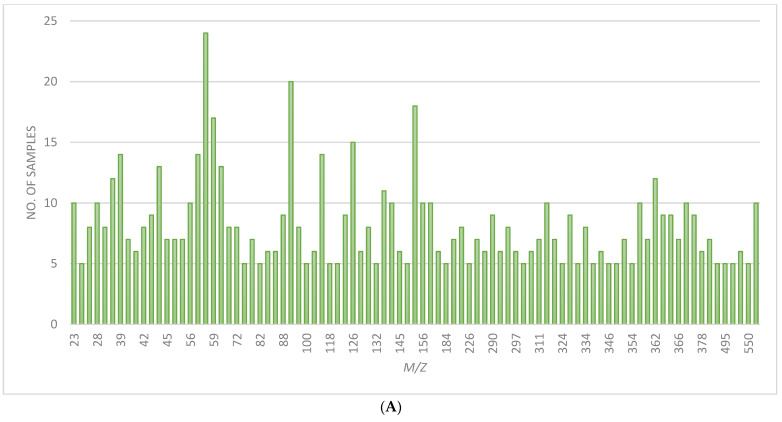

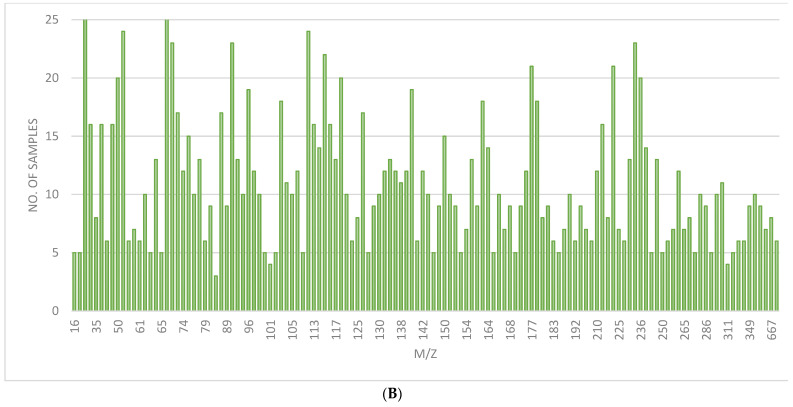
(**A**) Chart showing the no. of samples where ion images from positive mode ToF-SIMS showed ridge detail for common *m*/*z* values. (**B**) Chart showing the no. of samples where ion images from negative mode ToF-SIMS showed ridge detail for common *m*/*z* values.

Figure 4A,B shows the frequency of *m*/*z* values that provided fingerprint ridge detail. These varied contributions of ions to the images show how the multiplexed detection of analytes in fingerprints afforded by mass spectrometry offers an advantage over any method that targets a specific fingerprint constituent. It also means the generation of the best fingerprint image can be quite labour-intensive, and so, as shown in Charlton et al., multivariate analysis could be a useful tool to streamline the process if a standard protocol could be developed [16].

### 2.2. Limitations

As noted in Charlton et al., ToF-SIMS took just under 37 min to image an area of 6 × 6 mm. A fingerprint covering an area of 12 × 18 mm would therefore take ToF-SIMS 3 h 40 min to image under the same imaging conditions. This is lengthy compared to standard processes, so ToF-SIMS would probably only be applied to enhance insufficient fingermarks developed by standard processes. It also takes approximately 2 h (surface dependent) to reach sufficient vacuum in the chamber after loading the samples. The system also limits the size of an exhibit that could be examined at one time, in this case, to 10 × 7 cm, and only one side could be examined at a time.

In this work, the assessment of whether ToF-SIMS had enhanced the fingerprint was only carried out by one fingerprint expert. They were not involved in the research and were not expected to carry any bias. However, the use of a second or third fingerprint expert may provide greater confidence in the decision. It was not possible to specify exactly the area where the ToF-SIMS imaging was applied, but the fingerprint expert was asked to compare the centre of the image with the standard process and use identifying features where possible. It should be noted that the fingerprints for each variable were deposited at different times, which may lead to variability in composition.

This work shows ToF-SIMS applied after one (BPS) or two (CA and By40) standard processes. Full sequential treatment could involve more processes, such as powdering and vacuum metal deposition, so further work could look at the impact of more sequential processing on ToF-SIMS imaging. 

## 3. Materials and Methods

### 3.1. Fingerprint Deposition

**Figure 5:**
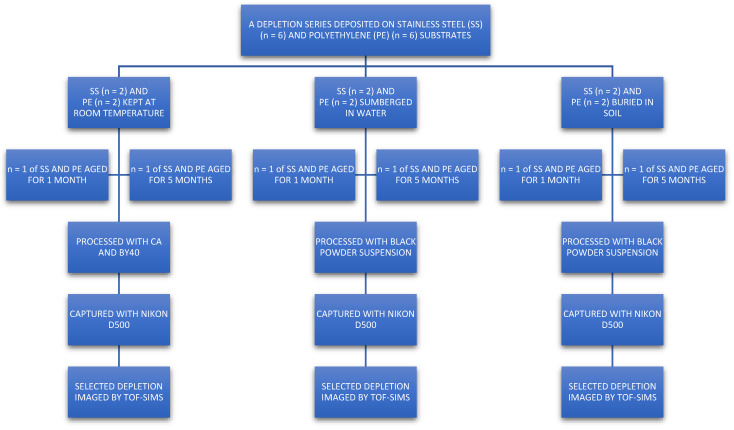
Schematic of workflow used for fingerprint deposition. Each donor (5 male, 5 female) used a different finger to deposit a depletion series (up to n = 88) for each substrate (PE = polyethylene, SS = stainless steel) and storage condition. This yielded 120 fingerprint sets, which were processed with Category A techniques. A fingerprint from each set was selected and then subsequently imaged using ToF-SIMS.

Natural “ungroomed” (no prescribed preparation such as hand washing or loading with secretions) fingerprints were used to represent fingermarks on crime scene exhibits best. Fingerprints from ten donors (five male, five female) were deposited (using six different fingers) on two substrates representative of common crime scene exhibits. Polyethylene was used to represent hard plastic packaging, and stainless steel to represent knives. As recommended by Holder et al., a depletion series was used to gradually decrease the amount of fingerprint residue left on the substrate surface. This was carried out to simulate fingerprints that are harder to visualise with standard processes, yielding poorer development [4,24]. Fingerprints were laid in a depletion series (using the same finger sequentially without reloading) up to n = 88. Each donor laid a depletion series on 6 samples of each substrate type. These were then stored for either 1 month or 5 months in either (a) rainwater, (b) 30 cm underground (burial), or (c) under ambient conditions (room temperature). After ageing, samples were treated with FVM-recommended processes [2]. A schematic of the workflow is shown in Figure 5, and this was repeated for each of the 10 donors. Each donor used a different finger for each depletion series they donated.

### 3.2. Cyanoacrylate Fuming and Basic Yellow 40 Dye Staining

Polyethylene and stainless steel samples that were stored dry at room temperature were developed with cyanoacrylate (industrial grade with minimal additives, Scenesafe, Burnham-on-Crouch, UK) using a Foster & Freeman MVC 3000 cabinet (Foster and Freeman, Evesham, UK). The cabinet is maintained within an ISO17025-accredited police laboratory, and the process was conducted according to FVM guidelines and the police laboratory’s standard operating procedures (SOPs). The cyanoacrylate was heated to 120 °C, and fuming was carried out at 80% RH. Each processing run is set up with a piece of plastic planted with a fingerprint loaded with amino acids using an amino acid-based latent print reference pad (Safariland, Jacksonville, FL, USA). This forms part of the quality control in the laboratory, and development was monitored and halted when the reference fingerprint reached optimum development. The developed samples were then immersed in a working solution of ethanol (96%, Acota, Oswestry, UK) and Basic Yellow 40 dye (>80%, Scenesafe, UK), according to laboratory SOPs. Samples were gently rinsed in running tap water and dried at 30 °C inside a drying cabinet. All samples were captured using a Nikon D500 camera with a 476 nm filter and crime-lite© 420–470 nm fluorescence illumination.

### 3.3. Black Powder Suspension

Polyethylene and stainless steel samples aged underwater or buried were developed using an iron (III) oxide black powder suspension (iron (III) oxide, tween 20, distilled water). Following FVM guidelines and laboratory SOPS, the surface is gently prewetted with tap water, and then the powder suspension is applied using a squirrely hair brush before rinsing off again with tap water. Samples were then dried at 30 °C inside a drying cabinet. All samples were captured using a Nikon D500 camera. Prior to processing the samples, a piece of white semiporous card planted with a fingerprint loaded with sebaceous oils using a sebaceous oil-based latent print reference pad (Safariland, Jacksonville, FL, USA) was successfully processed as above. This control test forms part of the quality control in the laboratory.

### 3.4. ToF-SIMS Imaging

ToF-SIMS imaging was carried out on one selected fingerprint from each of the 120 depletion series after the application of standard processes. The fingerprints selected were either the least developed in the series or entirely undeveloped by standard processes. The imaging area was aimed at the centre of the substrate to maximise the ridge density.

Analyses were carried out on an IonToF GmbH (Münster, Germany) ToF-SIMS 5 instrument, using a 25 keV Bi_3_^+^ primary ion beam delivering 0.18 pA of current, operating in the high current bunched mode. Raw data sets of total ion images were acquired at 600 × 600 pixels resolution in the raster (sawtooth) mode of operation. From these, mass-selected images were constructed. These parameters were selected as they provide good image resolution within an acceptable time frame for 6 mm × 6 mm image size, i.e., 36 min 48 s per image acquisition. Superior-quality images can be acquired by employing higher resolutions; however, these require, respectively, longer acquisition times [14]. Run settings were 10 frames per patch, 0.25 mm patch side length, 5 shots per pixel, 100 pixel density/mm, and 1 scan. These patches were then stitched together within the IonToF software (Surface Lab 6) to provide macroimages. 

Data analysis was performed using IonToF Surface Lab 6 software. After spectral calibration, a peak search, filtering by signal-to-noise ratio and peak intensity to around 350 peaks, was used to generate ion images. These were manually examined and combined to give the best image of fingerprint ridge detail, either using a signal from the fingerprint ridges or the background in contrast to the ridges. All ToF-SIMS images were normalised to the total ion current (TIC) to correct for fluctuations in ion beam current and differences in geometry as well as improve the quality of ridge detail. The exceptions were where ridge detail was better without normalisation.

### 3.5. Scoring Fingerprints

Images generated by standard processes were compared to images generated by sequential imaging of the samples by ToF-SIMS. This was carried out by a reporting fingerprint expert employed by Thames Valley Police (Kidlington, UK). A scoring system was used to report and compare results, whereby a score of 1 corresponded to improved ridge detail using ToF-SIMS (in either positive or negative more), and 0 corresponded to no improvement. As fingerprints were imaged in both positive and negative modes, where both modes yielded ridge detail, the reporting fingerprint expert was asked to assess which yielded the better image of the ridge detail. The ToF-SIMS sampled only a 6 × 6 mm area to increase throughput and, therefore, the comparison of ridge detail is only valid for the area imaged.

## 4. Conclusions

To our knowledge, this is the first large-scale study to test the general applicability of using ToF-SIMS for fingerprint development. We have shown that ToF-SIMS imaging was able to enhance fingerprint ridge detail further than the standard process for the majority of the 120 fingerprints tested here, even revealing ridge detail where the standard process failed to develop any. This shows that ToF-SIMS has the potential to fill some large gaps in the sequential fingerprint development process. If these were live casework examples, based on operational experience, many fingerprints would have gone undetected. When there is no ridge detail visualised by the standard process, there is no indicator to focus on ToF-SIMS as so it may not be a useful tool in these cases.

Using ToF-SIMS after standard processes proved successful and provided further enhancement on both substrates and across all exposure conditions. This expanded study has shown that ToF-SIMS would be suitable for targeted specialised fingermark enhancement on flat polyethylene and stainless steel surfaces aged for up to 5 months and exposed to ambient conditions, water or buried underground.

## Figures and Tables

**Table 1:** Selected images showing the different ions making up the ToF-SIMS images and how poor CA and BY40 development can result in better SIMS images.

	CA and BY40 Image	ToF-SIMS Positive Image	ToF-SIMS Negative Image
Substrate: PE Condition: Ambient Age: 5 Months Donor: M3 Deposition: 76	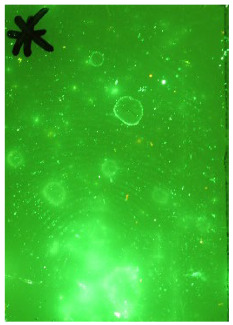	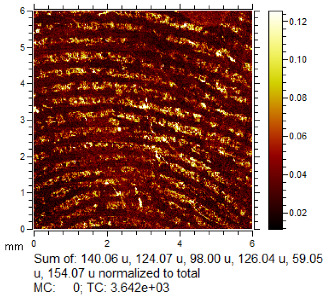	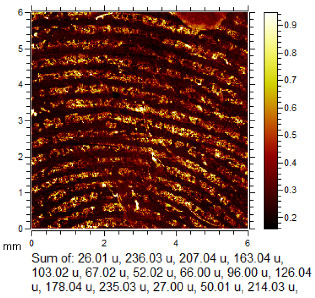
Substrate: SS Condition: Ambient Age: 5 Months Donor: M3 Deposition: 21	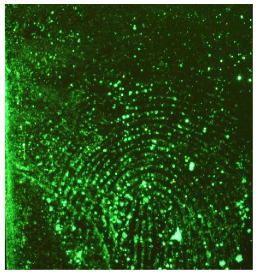	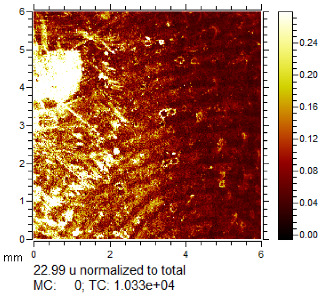	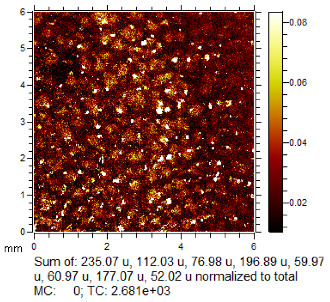

**Table 2:** Selected images showing examples of fingerprints assigned a “ToF-SIMS Enhancement Score” of 1, meaning the ToF-SIMS image provided clearer ridge detail than the standard process, as determined by a fingerprint expert working for Thames Valley Police.

	Standard Process Image	ToF-SIMS Image
Substrate: PE Condition: Soil Age: 1 Month Donor: M2 Deposition: 66	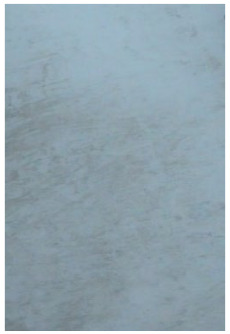 BPS	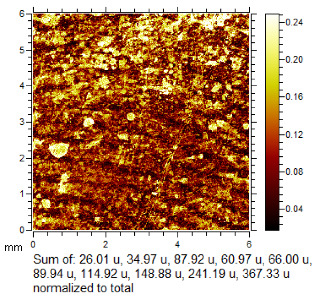 Negative Mode
Substrate: SS Condition: Water Age: 1 Month Donor: M4 Deposition: 1	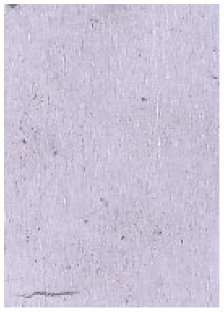 BPS	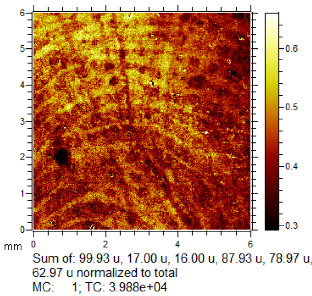 Negative Mode
Substrate: PE Condition: Soil Age: 5 Months Donor: F3 Deposition: 1	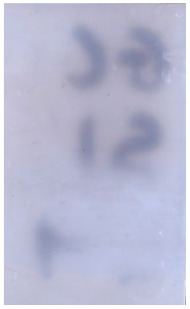 BPS	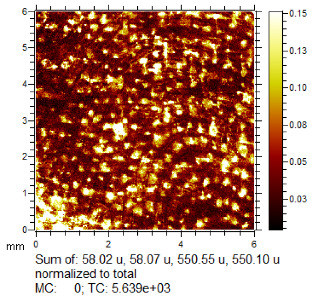 Positive Mode
Substrate: PE Condition: Ambient Age: 5 Months Donor: F5 Deposition: 26	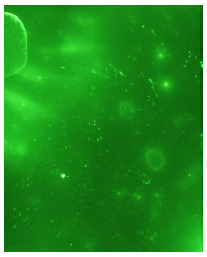 CA and BY40	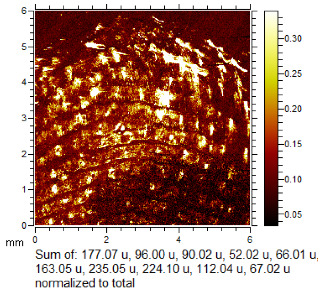 Negative Mode

## Data Availability

The data presented in this study are available on request from the corresponding author. The data are not publicly available due to privacy.

## Supplementary Materials

The following supporting information can be downloaded at: https://www.mdpi.com/article/10.3390/molecules28155687/s1, Table S1: Images of ridge detail of all donors on stainless-steel and polyethylene visualised by standard developers (cyanoacrylate and basic yellow 40 or black powder suspension) and ToF-SIMS (positive or negative mode) after aging for 1 month or 5 months under all conditions (ambient, soil or water). X signifies there were no ions that were found to yield an image of ridge detail.; Table S2: Table of common *m*/*z* values yielded from positive mode ToF-SIMS for male and female donors on polyethylene and stainless-steel after aging for 1 month or 5 months under all conditions (ambient, soil or water). The number indicates in how many donors the *m*/*z* value yielded an image of ridge detail, for each condition.; Table S3: Table of common *m*/*z* values yielded from negative mode ToF-SIMS for male and female donors on polyethylene and stainless-steel after aging for 1 month or 5 months under all conditions (ambient, soil or water). The number indicates in how many donors the *m*/*z* value yielded an image of ridge detail, for each condition.
