# Supplementary material for: Expanding the Efficacy of Fingermark Enhancement Using ToF-SIMS
Source: Molecules. 2023 Jul 27;28(15):5687. doi: 10.3390/molecules28155687 (PMC10419545; doi:10.3390/molecules28155687)
Supplement: Supplementary file 1 [file molecules-28-05687-s001.zip › molecules-2398992-supplementary.pdf]

Supporting Information Table S1 – Images of ridge detail of all donors on stainless-steel and polyethylene visualised by standard developers (cyanoacrylate and basic yellow 40 or black powder suspension) and ToF-SIMS (positive or negative mode) after aging for 1 month or 5 months under all conditions (ambient, soil or water). X signifies there were no ions that were found to yield an image of ridge detail.

	Standard Process Image	ToF-SIMS Positive Mode Image	ToF-SIMS Negative Mode Image
**Substrate:** Polyethylene **Condition:** Ambient **Age**: 1 Month **Donor**: F1 **Deposition:** 30	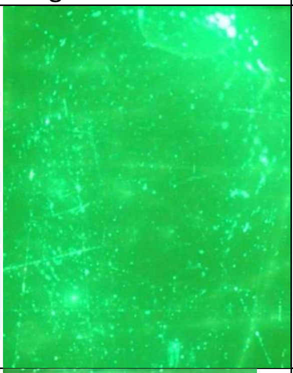	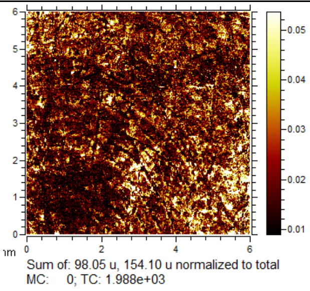	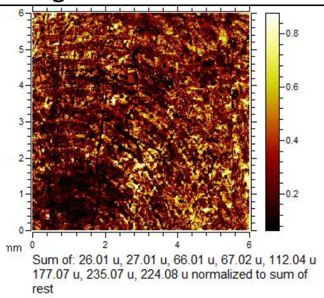
**Substrate:** Polyethylene **Condition:** Ambient **Age**: 1 Month **Donor**: F2 **Deposition:** 29	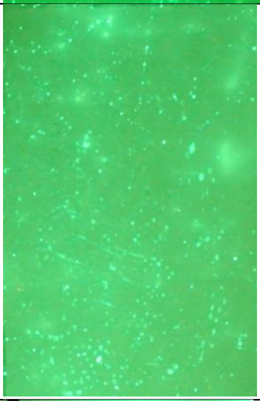	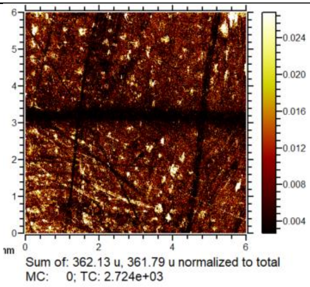	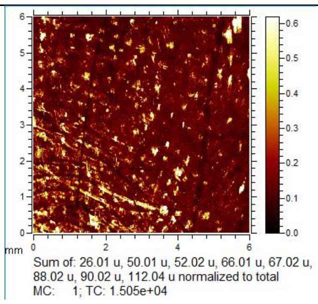
**Substrate:** Polyethylene **Condition:** Ambient **Age**: 1 Month **Donor**: F3 **Deposition:** 88	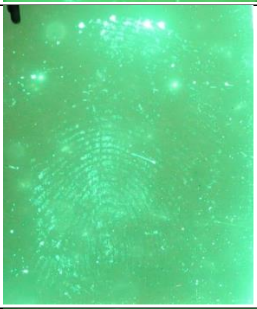	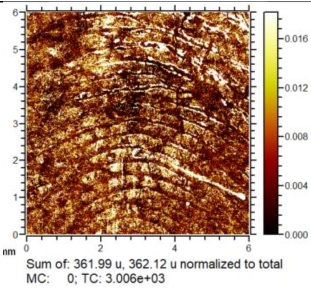	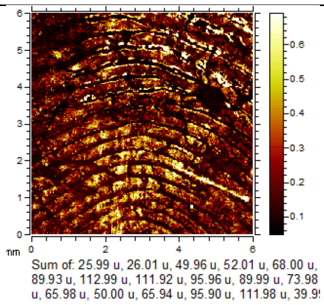
**Substrate:** Polyethylene **Condition:** Soil **Age**: 1 Month **Donor**: F4 **Deposition:** 20	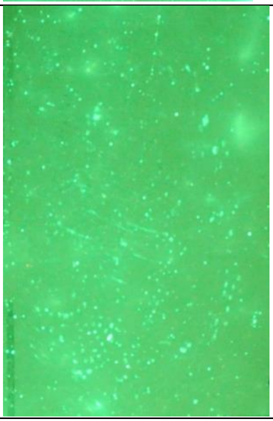	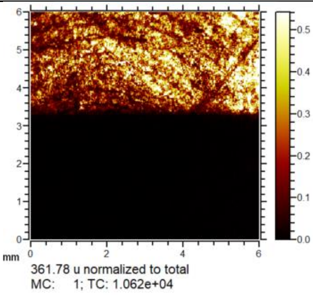	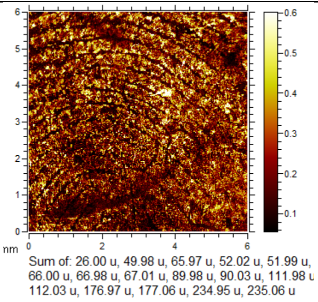

**Substrate:** Polyethylene<br>**Condition:** Ambient<br>**Age**: 1 Month<br>**Donor**: F5<br>**Deposition:** 23	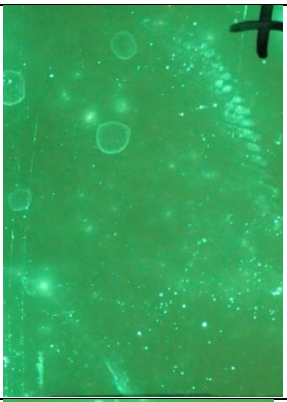	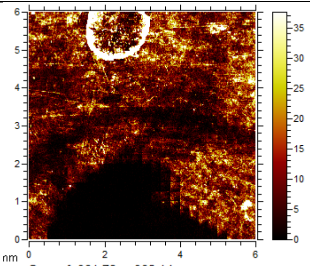	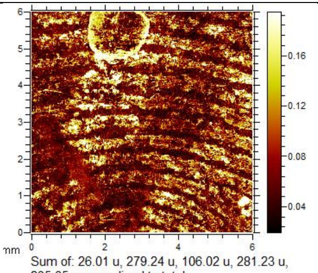
**Substrate:** Polyethylene<br>**Condition:** Ambient<br>**Age**: 1 Month<br>**Donor**: M1<br>**Deposition:** 74	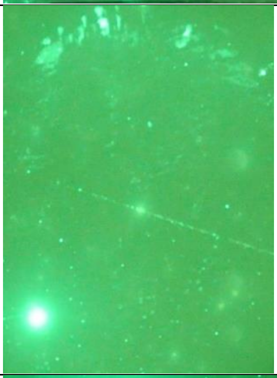	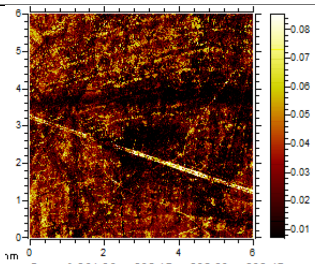	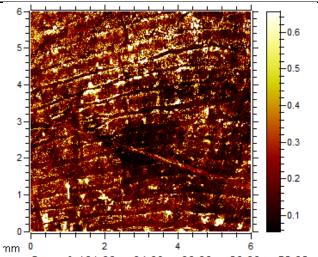
**Substrate:** Polyethylene<br>**Condition:** Ambient<br>**Age**: 1 Month<br>**Donor**: M2<br>**Deposition:** 87	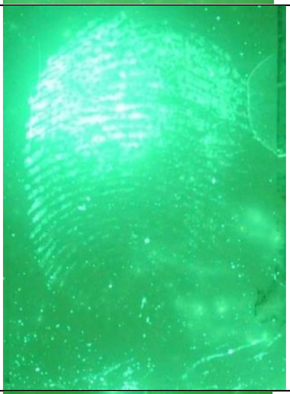	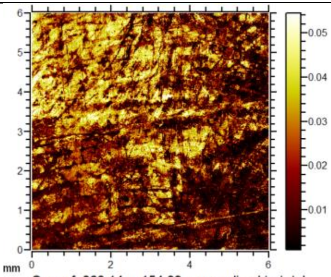	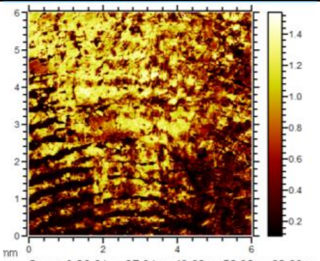
**Substrate:** Polyethylene<br>**Condition:** Ambient<br>**Age**: 1 Month<br>**Donor**: M3<br>**Deposition:** 46	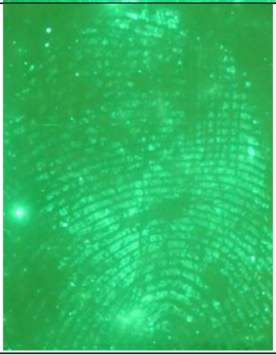	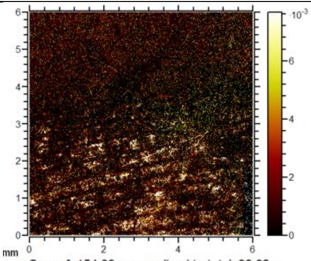	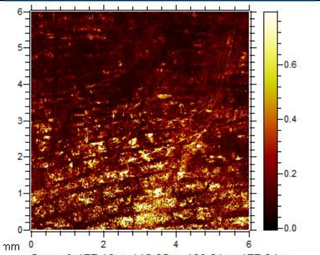

**Substrate:** Polyethylene<br>**Condition:** Ambient<br>**Age**: 1 Month<br>**Donor**: M4<br>**Deposition:** 87	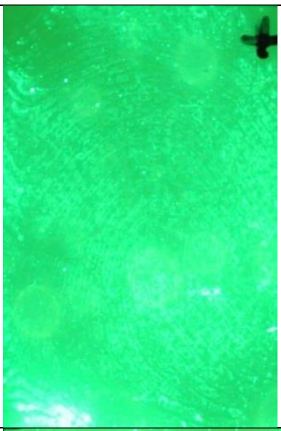	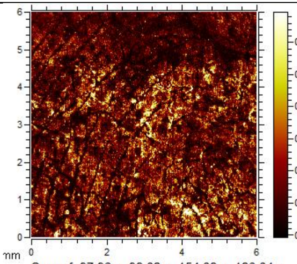<br>Sum of: 97.96 u, 98.02 u, 154.08 u, 126.04 u normalized to total<br>MC: 0; TC: 1.492e+03	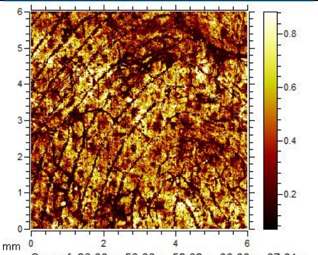<br>Sum of: 26.00 u, 50.00 u, 52.02 u, 66.00 u, 67.01 u, 90.02 u, 112.03 u, 163.04 u normalized to total<br>MC: 1; TC: 3.599e+04
**Substrate:** Polyethylene<br>**Condition:** Ambient<br>**Age**: 1 Month<br>**Donor**: M5<br>**Deposition:** 39	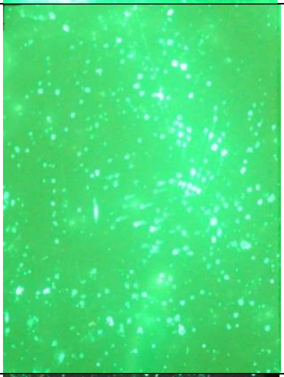	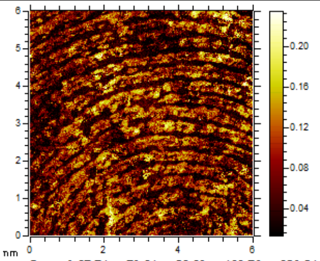<br>Sum of: 27.71 u, 70.34 u, 53.29 u, 188.70 u, 250.51 u, 71.42 u normalized to total<br>MC: 0; TC: 2.630e+04	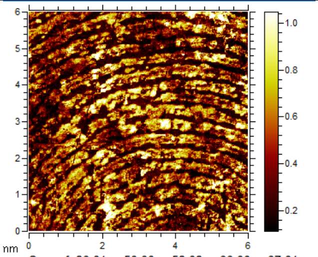<br>Sum of: 26.01 u, 50.00 u, 52.02 u, 66.00 u, 67.01 u, 88.01 u, 90.02 u, 96.00 u, 112.03 u, 163.05 u, 177.0[illegible] u, 224.06 u, 235.04 u normalized to total
**Substrate:** Stainless-steel<br>**Condition:** Ambient<br>**Age**: 1 Month<br>**Donor**: F1<br>**Deposition:** 47	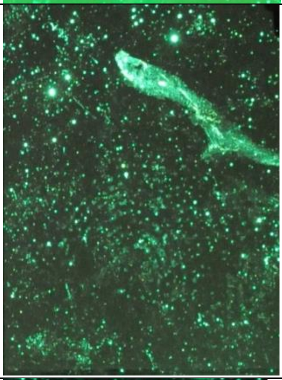	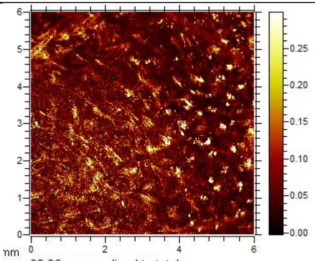<br>38.96 u normalized to total<br>MC: 0; TC: 6.786e+03	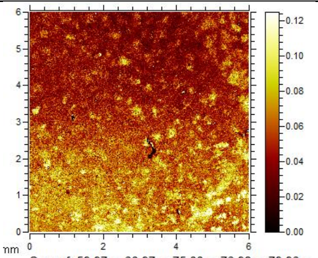<br>Sum of: 59.97 u, 60.97 u, 75.00 u, 76.98 u, 79.96 u, 183.02 u normalized to total<br>MC: 0; TC: 5.052e+03
**Substrate:** Stainless-steel<br>**Condition:** Ambient<br>**Age**: 1 Month<br>**Donor**: F2<br>**Deposition:** 20	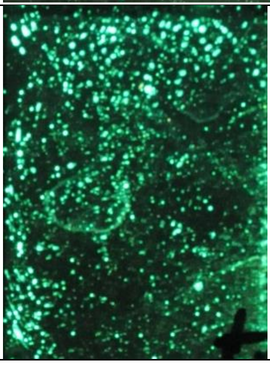	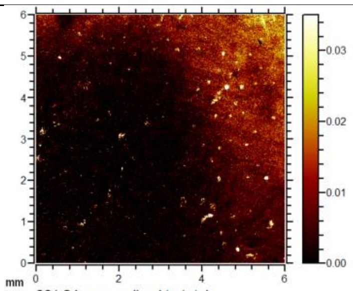<br>361.24 u normalized to total<br>MC: 0; TC: 1.626e+03	X

**Substrate:** Stainless-steel<br>**Condition:** Ambient<br>**Age**: 1 Month<br>**Donor**: F3<br>**Deposition:** 60	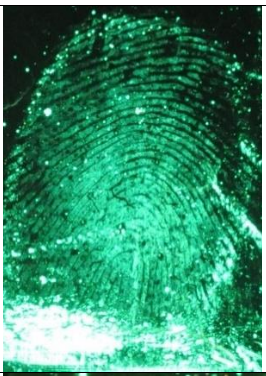	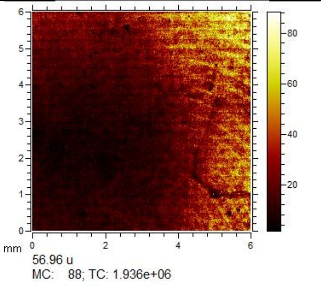	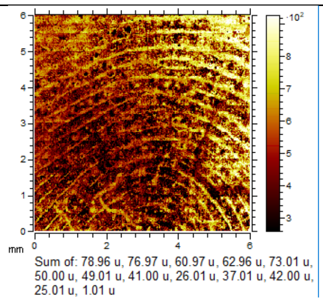
**Substrate:** Stainless-steel<br>**Condition:** Ambient<br>**Age**: 1 Month<br>**Donor**: F4<br>**Deposition:** 20	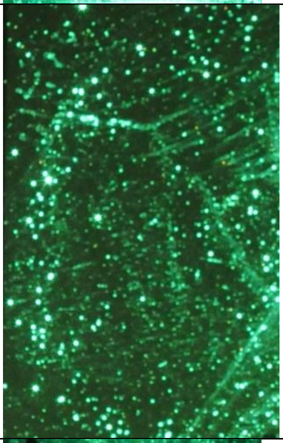	X	X
**Substrate:** Stainless-steel<br>**Condition:** Ambient<br>**Age**: 1 Month<br>**Donor**: F5<br>**Deposition:** 1	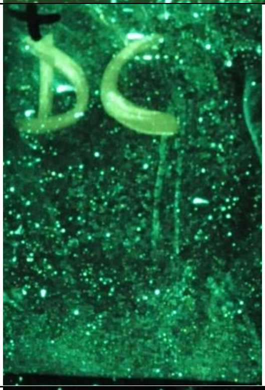	x	X
**Substrate:** Stainless-steel<br>**Condition:** Ambient<br>**Age**: 1 Month<br>**Donor**: M1<br>**Deposition:** 60	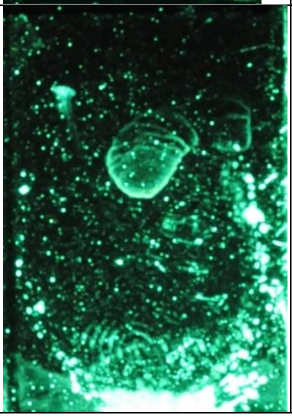	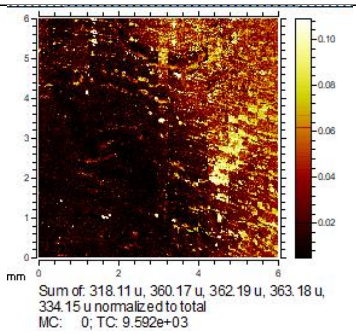	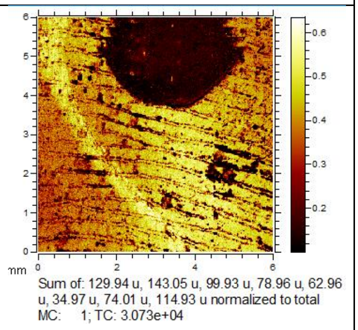

**Substrate:** Stainless-steel **Condition:** Ambient **Age**: 1 Month **Donor**: M2 **Deposition:** 60	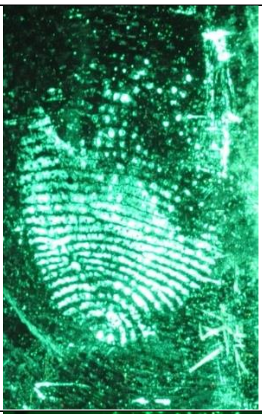	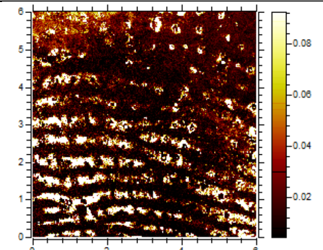	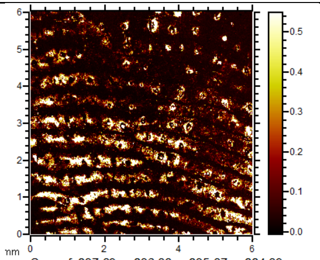
**Substrate:** Stainless-steel **Condition:** Ambient **Age**: 1 Month **Donor**: M3 **Deposition:** 22	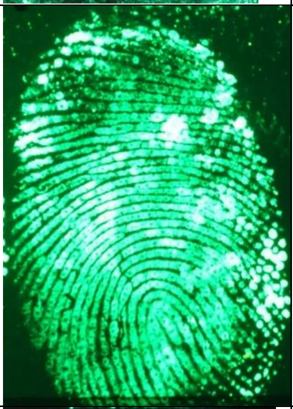	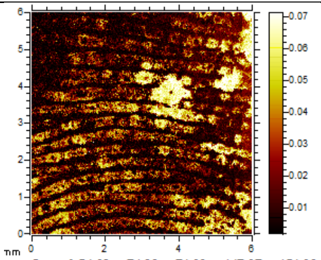	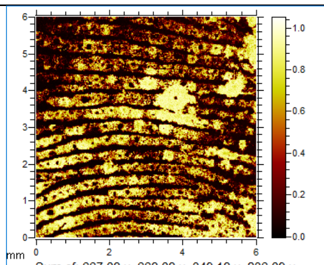
**Substrate:** Stainless-steel **Condition:** Ambient **Age**: 1 Month **Donor**: M4 **Deposition:** 59	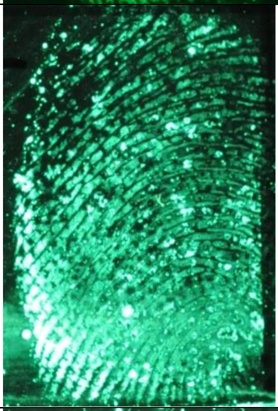	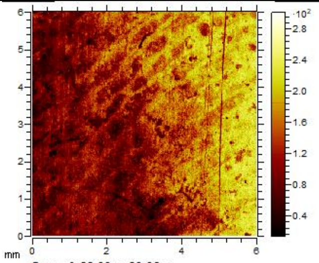	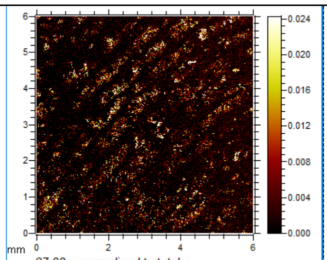
**Substrate:** Stainless-steel **Condition:** Ambient **Age**: 1 Month **Donor**: M5 **Deposition:** 15	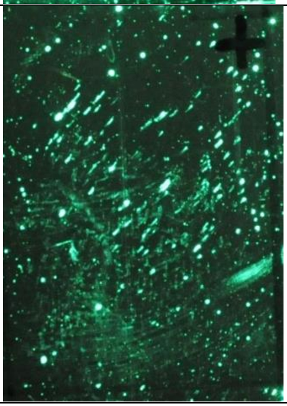	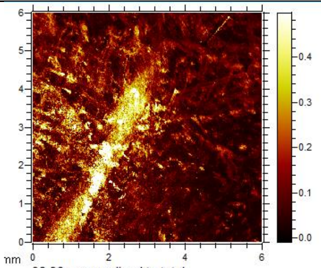	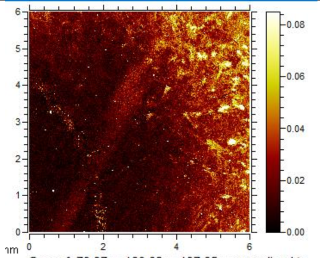

**Substrate:** Polyethylene<br>**Condition:** Soil<br>**Age**: 1 Month<br>**Donor**: F1<br>**Deposition:** 30	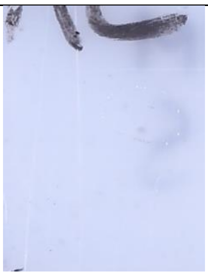	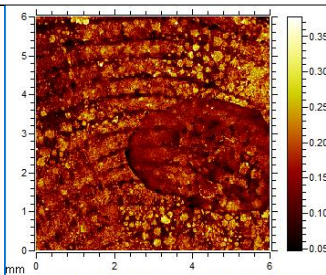 	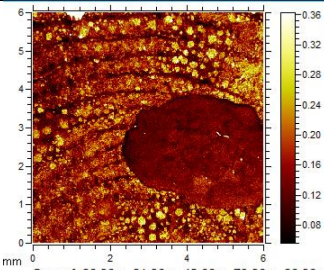
**Substrate:** Polyethylene<br>**Condition:** Soil<br>**Age**: 1 Month<br>**Donor**: F2<br>**Deposition:** 1	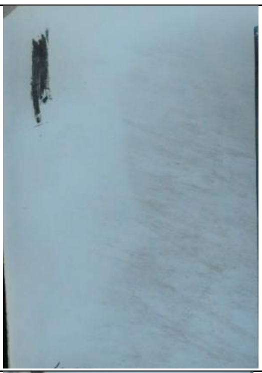	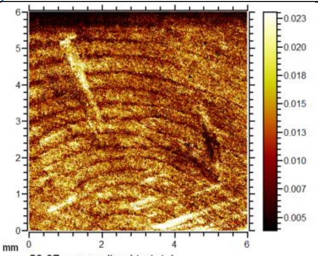 	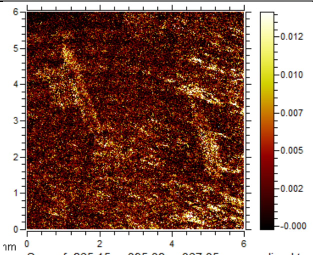
**Substrate:** Polyethylene<br>**Condition:** Soil<br>**Age**: 1 Month<br>**Donor**: F3<br>**Deposition:** 1	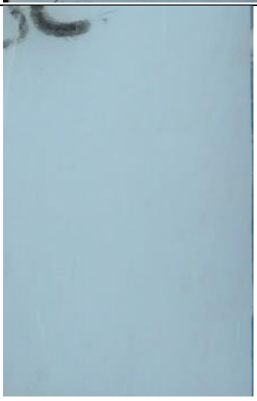	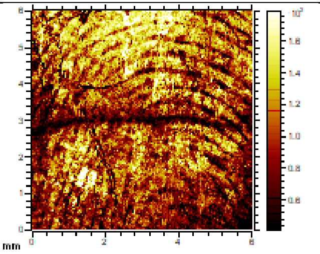<br><br>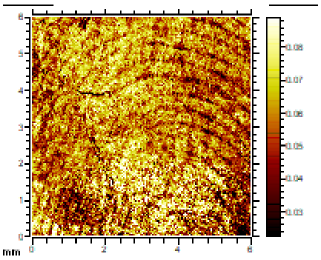 	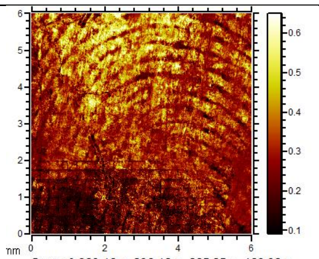
**Substrate:** Polyethylene<br>**Condition:** Soil<br>**Age**: 1 Month<br>**Donor**: F4<br>**Deposition:** 1	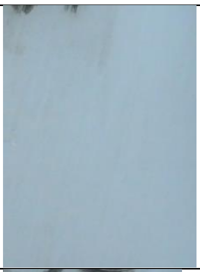	x	x
**Substrate:** Polyethylene<br>**Condition:** Soil<br>**Age**: 1 Month<br>**Donor**: F5<br>**Deposition:** 1	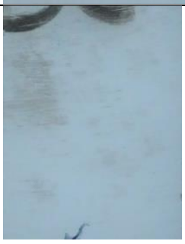	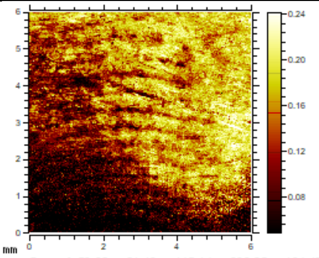 	X

**Substrate:** Polyethylene<br>**Condition:** Soil<br>**Age**: 1 Month<br>**Donor**: M1<br>**Deposition:** 1	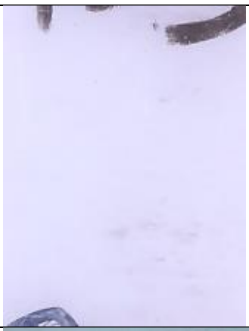	X	X
**Substrate:** Polyethylene<br>**Condition:** Soil<br>**Age**: 1 Month<br>**Donor**: M2<br>**Deposition:** 66	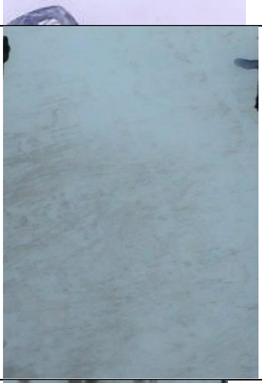	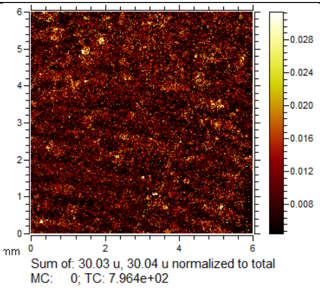	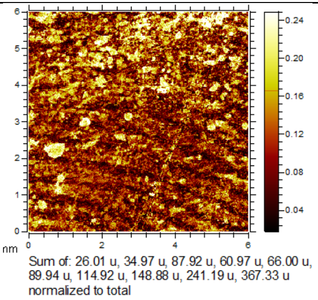
**Substrate:** Polyethylene<br>**Condition:** Soil<br>**Age**: 1 Month<br>**Donor**: M3<br>**Deposition:** 1	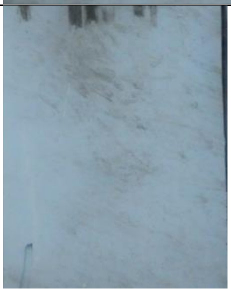	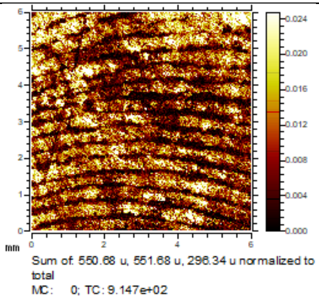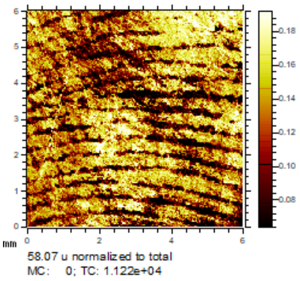	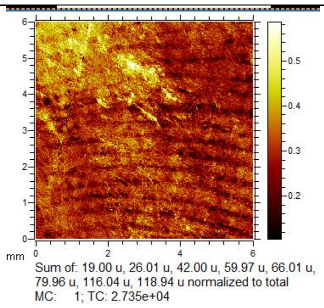
**Substrate:** Polyethylene<br>**Condition:** Soil<br>**Age**: 1 Month<br>**Donor**: M4<br>**Deposition:** 7	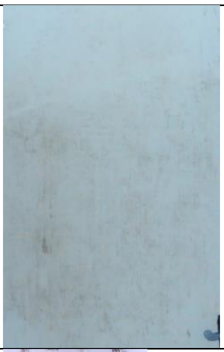	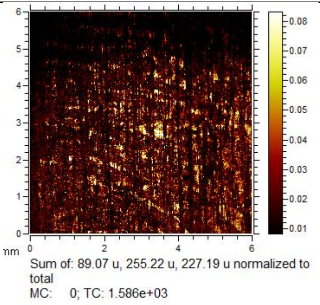	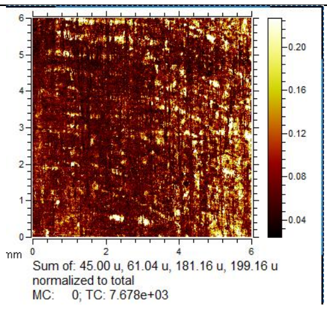
**Substrate:** Polyethylene<br>**Condition:** Soil<br>**Age**: 1 Month<br>**Donor**: M5<br>**Deposition:** 15	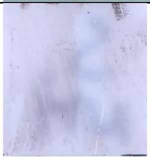	X	X

**Substrate:** Stainless-steel<br>**Condition:** Soil<br>**Age**: 1 Month<br>**Donor**: F1<br>**Deposition:** 1	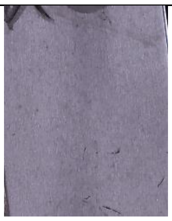	X	X
**Substrate:** Stainless-steel<br>**Condition:** Soil<br>**Age**: 1 Month<br>**Donor**: F2<br>**Deposition:** 20	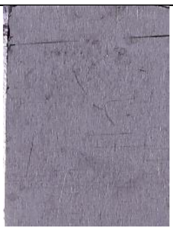	X	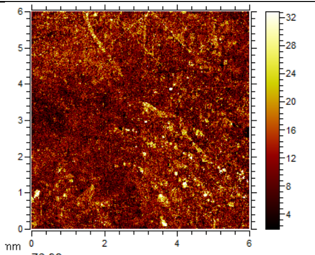<br>76.98 u<br>MC: 33; TC: 9.653e+05
**Substrate:** Stainless-steel<br>**Condition:** Soil<br>**Age**: 1 Month<br>**Donor**: F3<br>**Deposition:** 60	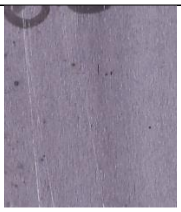	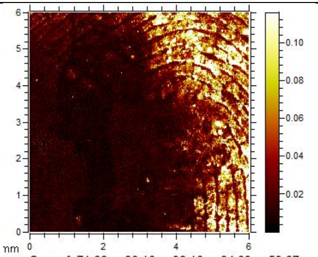<br>Sum of: 71.08 u, 86.10 u, 98.10 u, 84.09 u, 59.07 u, 45.06 u, 46.07 u, 58.07 u, 44.05 u, 42.03 u normalized to total	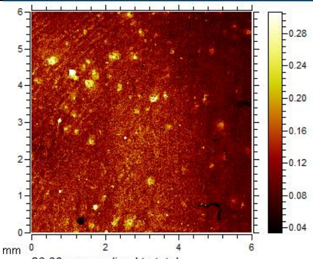<br>26.00 u normalized to total<br>MC: 0; TC: 1.072e+04
**Substrate:** Stainless-steel<br>**Condition:** Soil<br>**Age**: 1 Month<br>**Donor**: F4<br>**Deposition:** 1	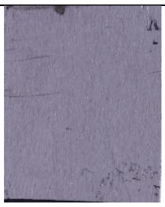	X	X
**Substrate:** Stainless-steel<br>**Condition:** Soil<br>**Age**: 1 Month<br>**Donor**: F5<br>**Deposition:** 2	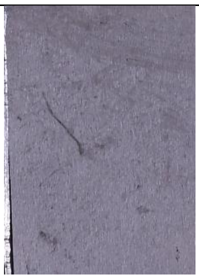	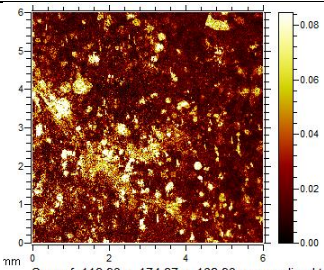<br>Sum of: 118.93 u, 174.87 u, 102.93 u normalized to total<br>MC: 0; TC: 2.381e+03	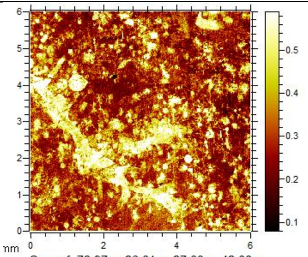<br>Sum of: 78.97 u, 26.01 u, 27.03 u, 42.00 u normalized to total<br>MC: 1; TC: 3.084e+04
**Substrate:** Stainless-steel<br>**Condition:** Soil<br>**Age**: 1 Month<br>**Donor**: M1<br>**Deposition:** 1	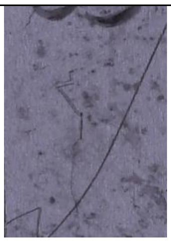	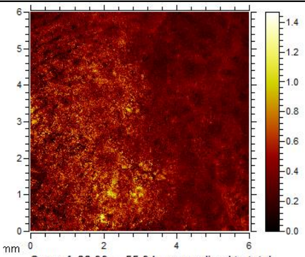<br>Sum of: 22.99 u, 55.94 u normalized to total<br>MC: 1; TC: 3.399e+04	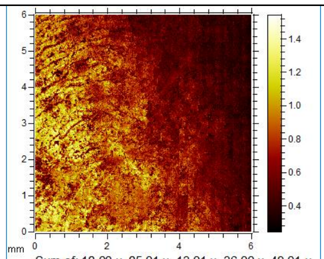<br>Sum of: 12.00 u, 25.01 u, 13.01 u, 36.00 u, 49.01 u normalized to total<br>MC: 2; TC: 6.337e+04

**Substrate:** Stainless-steel<br>**Condition:** Soil<br>**Age**: 1 Month<br>**Donor**: M2<br>**Deposition:** 60	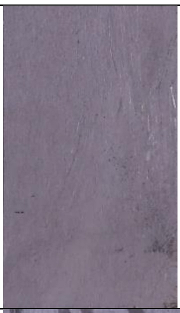	X	X
**Substrate:** Stainless-steel<br>**Condition:** Soil<br>**Age**: 1 Month<br>**Donor**: M3<br>**Deposition:** 1	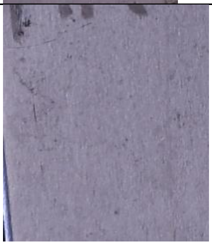	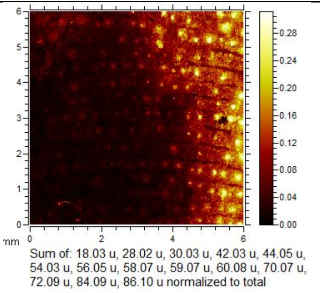	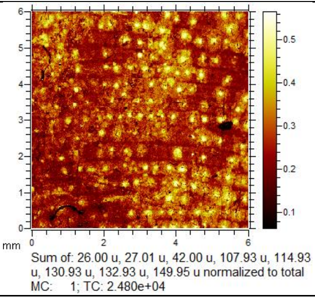
**Substrate:** Stainless-steel<br>**Condition:** Soil<br>**Age**: 1 Month<br>**Donor**: M4<br>**Deposition:** 1	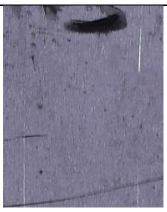	X	X
**Substrate:** Stainless-steel<br>**Condition:** Soil<br>**Age**: 1 Month<br>**Donor**: M5<br>**Deposition:** 1	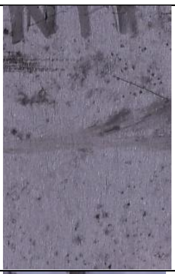	X	X
**Substrate:** Polyethylene<br>**Condition:** Water<br>**Age**: 1 Month<br>**Donor**: F1<br>**Deposition:** 1	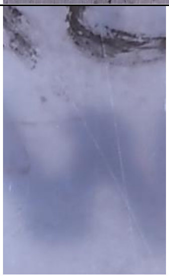	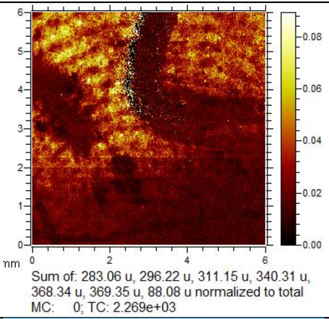	X
**Substrate:** Polyethylene<br>**Condition:** Water<br>**Age**: 1 Month<br>**Donor**: F2<br>**Deposition:** 1	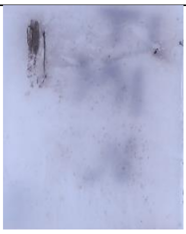	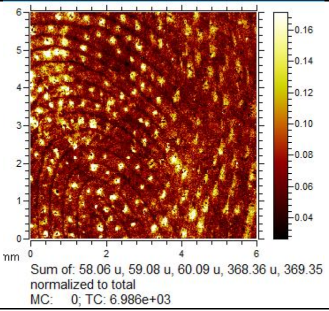	X

**Substrate:** Polyethylene<br>**Condition:** Water<br>**Age**: 1 Month<br>**Donor**: F3<br>**Deposition:** 1	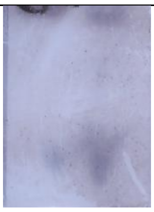	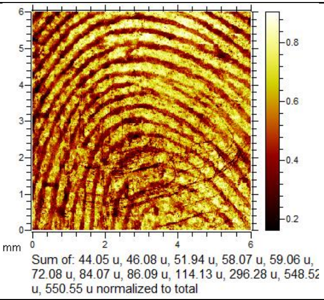	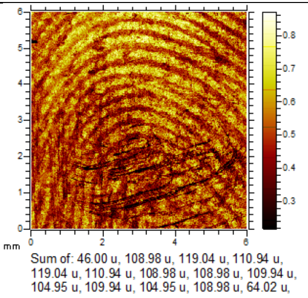
**Substrate:** Polyethylene<br>**Condition:** Water<br>**Age**: 1 Month<br>**Donor**: F4<br>**Deposition:** 1	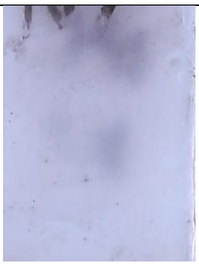	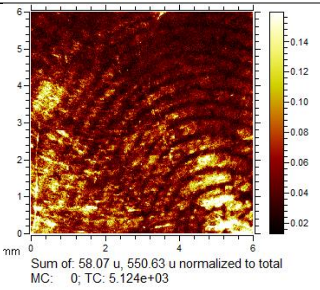	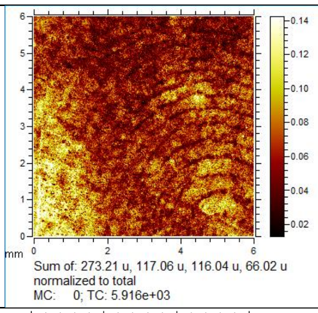
**Substrate:** Polyethylene<br>**Condition:** Water<br>**Age**: 1 Month<br>**Donor**: F5<br>**Deposition:** 9	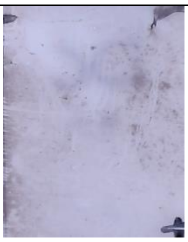	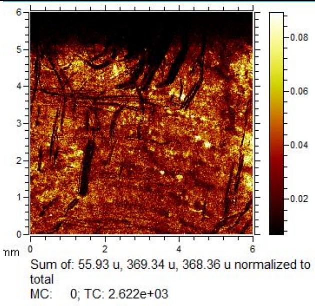	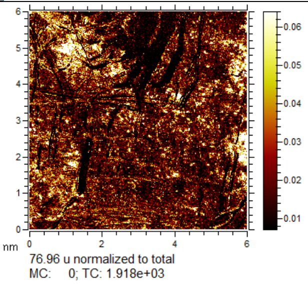
**Substrate:** Polyethylene<br>**Condition:** Water<br>**Age**: 1 Month<br>**Donor**: M1<br>**Deposition:** 5	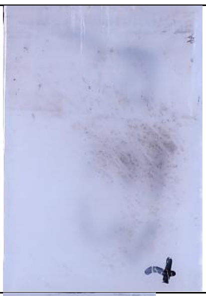	X	X
**Substrate:** Polyethylene<br>**Condition:** Water<br>**Age**: 1 Month<br>**Donor**: M2<br>**Deposition:** 6	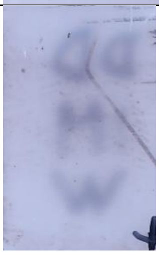	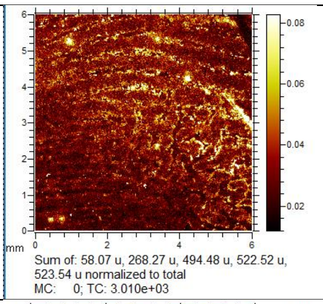	X
**Substrate:** Polyethylene<br>**Condition:** Water<br>**Age**: 1 Month<br>**Donor**: M3<br>**Deposition:** 1	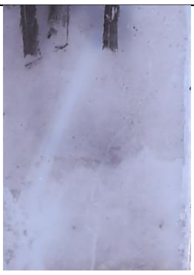	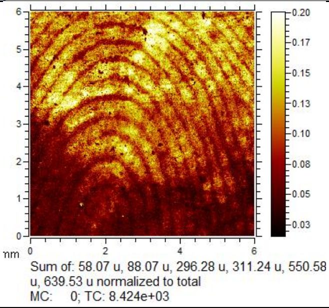	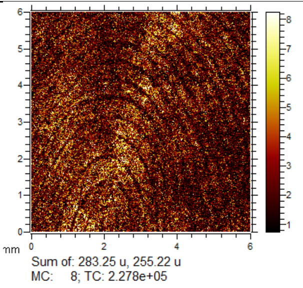

**Substrate:** Polyethylene<br>**Condition:** Water<br>**Age**: 1 Month<br>**Donor**: M4<br>**Deposition:** 5	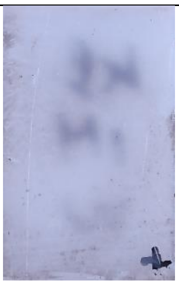	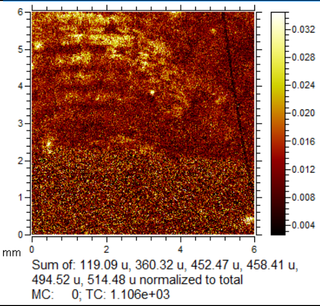	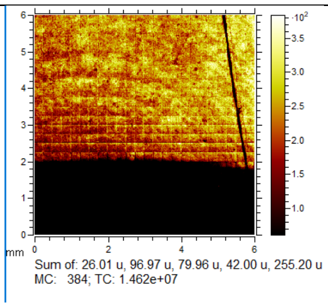
**Substrate:** Polyethylene<br>**Condition:** Water<br>**Age**: 1 Month<br>**Donor**: M5<br>**Deposition:** 7	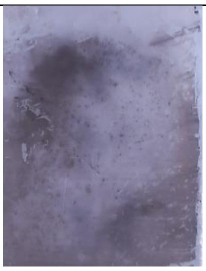	X	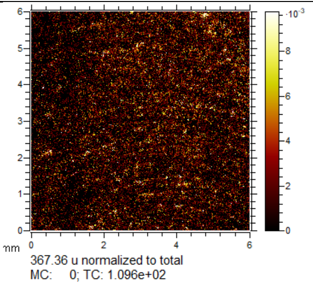
**Substrate:** Stainless-steel<br>**Condition:** Water<br>**Age**: 1 Month<br>**Donor**: F1<br>**Deposition:** 3	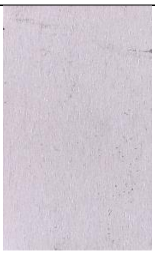	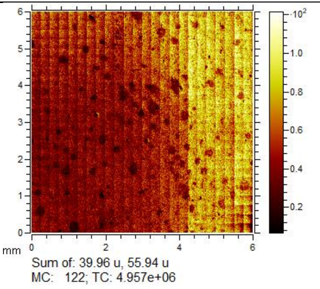	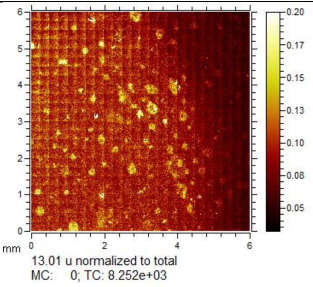
**Substrate:** Stainless-steel<br>**Condition:** Water<br>**Age**: 1 Month<br>**Donor**: F2<br>**Deposition:** 1	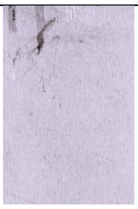	X	X
**Substrate:** Stainless-steel<br>**Condition:** Water<br>**Age**: 1 Month<br>**Donor**: F3<br>**Deposition:** 1	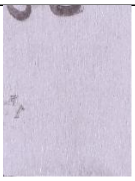	X	X
**Substrate:** Stainless-steel<br>**Condition:** Water<br>**Age**: 1 Month<br>**Donor**: F4<br>**Deposition:** 1	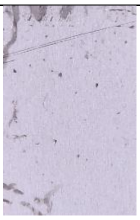	X	X

**Substrate:** Stainless-steel<br>**Condition:** Water<br>**Age**: 1 Month<br>**Donor**: F5<br>**Deposition:** 1	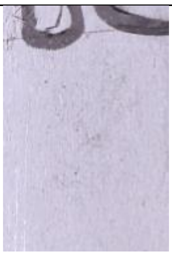	X	X
**Substrate:** Stainless-steel<br>**Condition:** Water<br>**Age**: 1 Month<br>**Donor**: M1<br>**Deposition:** 10	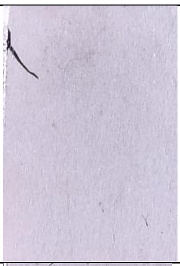	X	X
**Substrate:** Stainless-steel<br>**Condition:** Water<br>**Age**: 1 Month<br>**Donor**: M2<br>**Deposition:** 60	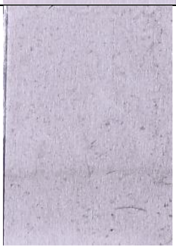	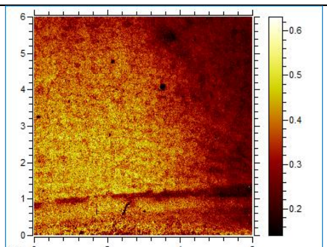	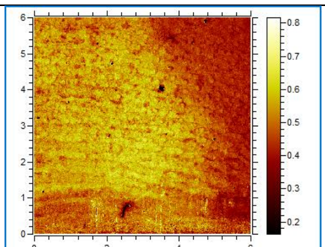
**Substrate:** Stainless-steel<br>**Condition:** Water<br>**Age**: 1 Month<br>**Donor**: M3<br>**Deposition:** 6	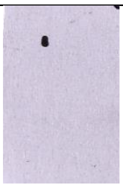	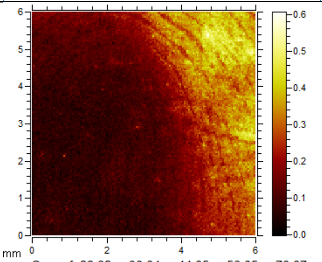	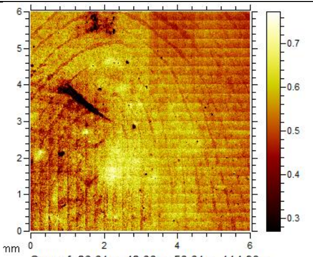
**Substrate:** Stainless-steel<br>**Condition:** Water<br>**Age**: 1 Month<br>**Donor**: M4<br>**Deposition:** 1	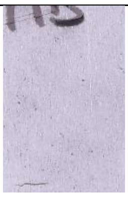	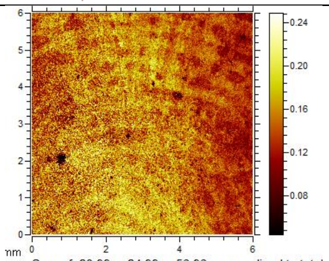	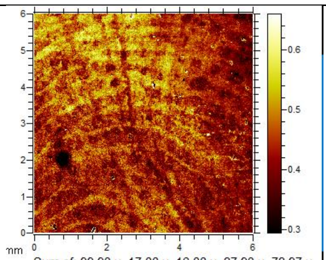
**Substrate:** Stainless-steel<br>**Condition:** Water<br>**Age**: 1 Month<br>**Donor**: M5<br>**Deposition:** 1	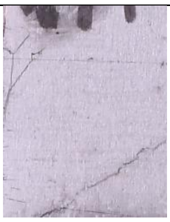	X	X

**Substrate:** Polyethylene<br>**Condition:** Ambient<br>**Age**: 5 Months<br>**Donor**: F1<br>**Deposition:** 86	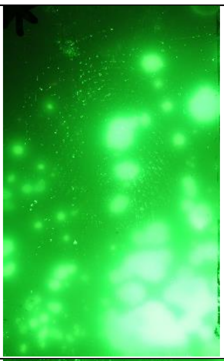	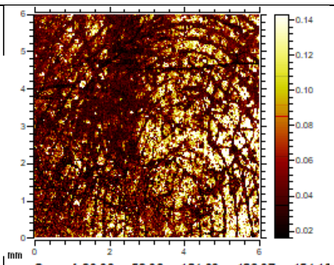	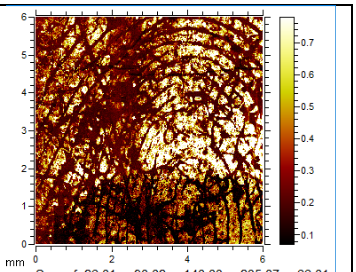
**Substrate:** Polyethylene<br>**Condition:** Ambient<br>**Age**: 5 Months<br>**Donor**: F2<br>**Deposition:** 21	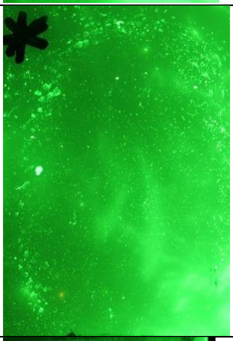	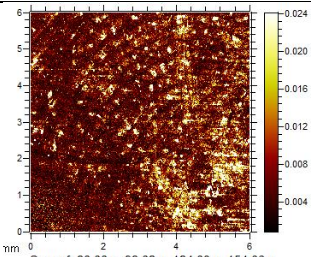	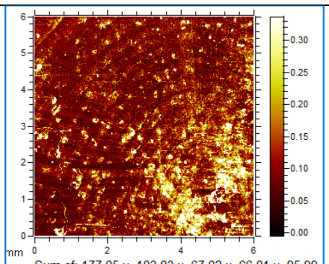
**Substrate:** Polyethylene<br>**Condition:** Ambient<br>**Age**: 5 Months<br>**Donor**: F3<br>**Deposition:** 88	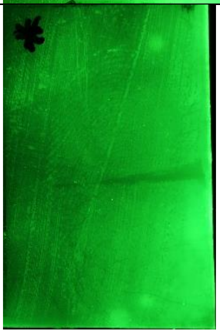	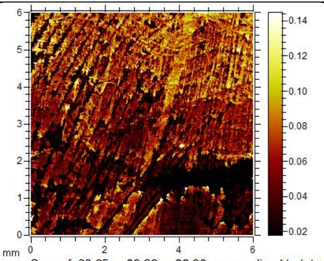	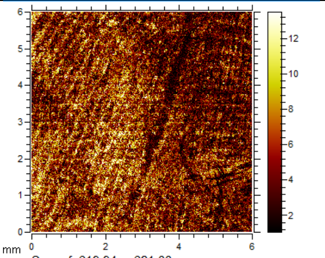<br>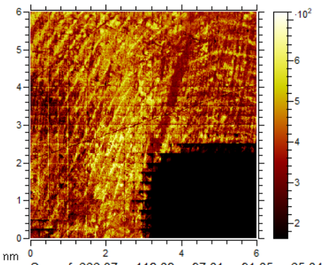
**Substrate:** Polyethylene<br>**Condition:** Ambient<br>**Age**: 5 Months<br>**Donor**: F4<br>**Deposition:** 64	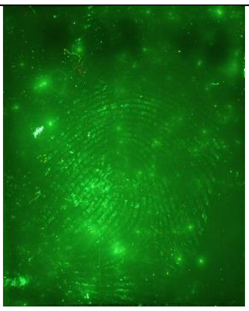	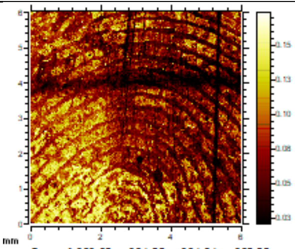	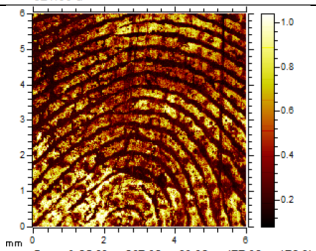

<table>
<tr><td>Substrate: Polyethylene
Condition: Ambient
Age: 5 Months
Donor: F5
Deposition: 26</td><td>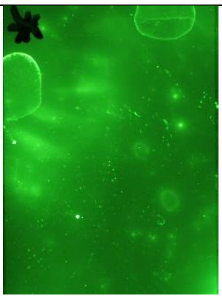</td><td>X</td><td>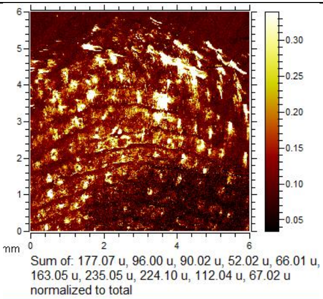
</td></tr>
<tr><td>Substrate: Polyethylene
Condition: Ambient
Age: 5 Months
Donor: M1
Deposition: 19</td><td>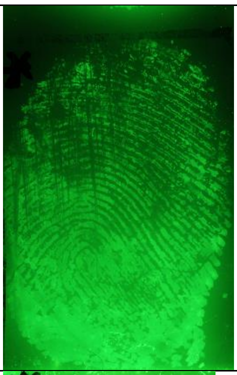</td><td>X</td><td>X</td></tr>
<tr><td>Substrate: Polyethylene
Condition: Ambient
Age: 5 Months
Donor: M2
Deposition: 1</td><td>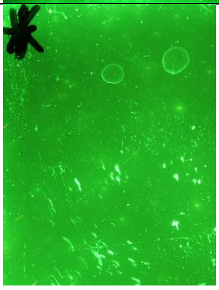</td><td>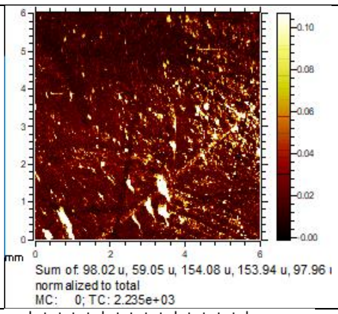
</td><td>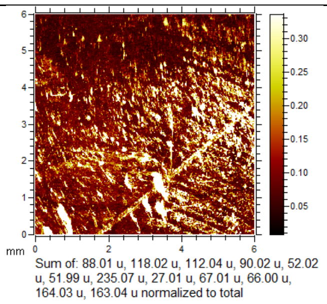
</td></tr>
<tr><td>Substrate: Polyethylene
Condition: Ambient
Age: 5 Months
Donor: M3
Deposition: 76</td><td>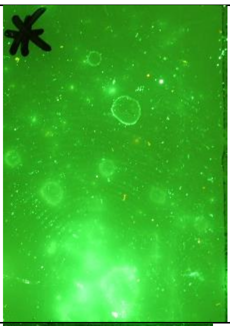</td><td>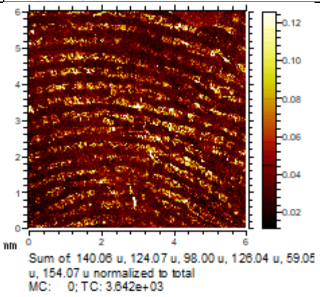
</td><td>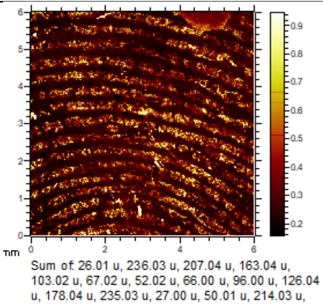
</td></tr>
<tr><td>Substrate: Polyethylene
Condition: Ambient
Age: 5 Months
Donor: M4
Deposition: 25</td><td>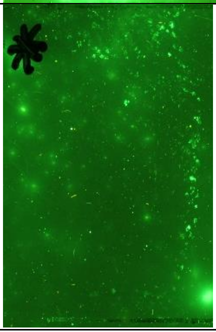</td><td>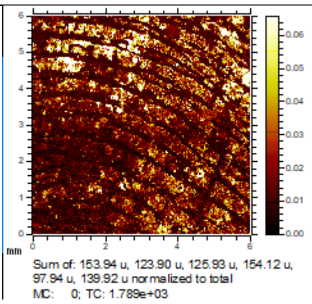
</td><td>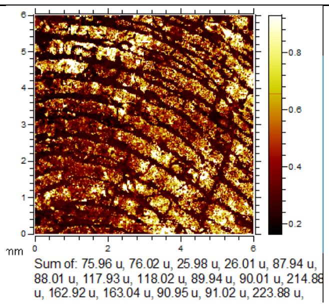
</td></tr>
</table>

<table>
<tr><td>Substrate: Polyethylene
Condition: Ambient
Age: 5 Months
Donor: M5
Deposition: 15</td><td>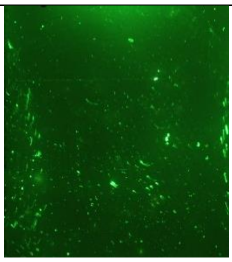</td><td>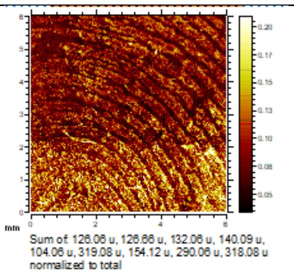
</td><td>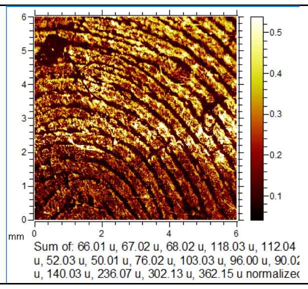
</td></tr>
<tr><td>Substrate: Stainless-steel
Condition: Ambient
Age: 5 Months
Donor: F1
Deposition: 70</td><td>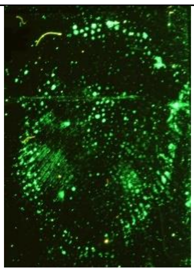</td><td>X</td><td>X</td></tr>
<tr><td>Substrate: Stainless-steel
Condition: Ambient
Age: 5 Months
Donor: F2
Deposition: 10</td><td>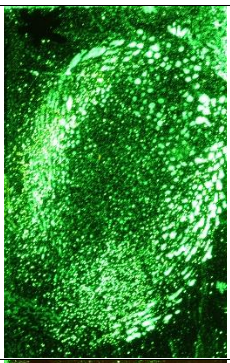</td><td>X</td><td>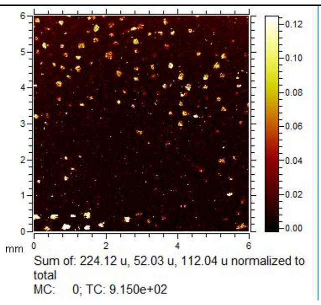
</td></tr>
<tr><td>Substrate: Stainless-steel
Condition: Ambient
Age: 5 Months
Donor: F3
Deposition: 71</td><td>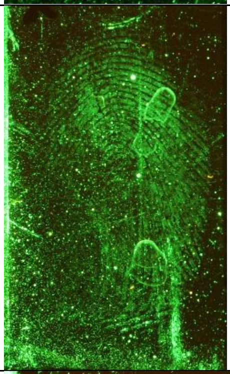</td><td>X</td><td>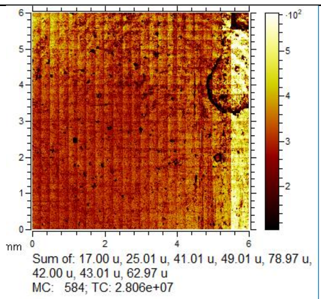
</td></tr>
<tr><td>Substrate: Stainless-steel
Condition: Ambient
Age: 5 Months
Donor: F4
Deposition: 41</td><td>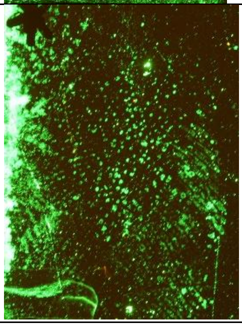</td><td>X</td><td>X</td></tr>
</table>

**Substrate:** Stainless-steel<br>**Condition:** Ambient<br>**Age**: 5 Months<br>**Donor**: F5<br>**Deposition:** 1	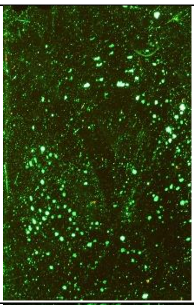	X	X
**Substrate:** Stainless-steel<br>**Condition:** Ambient<br>**Age**: 5 Months<br>**Donor**: M1<br>**Deposition:** 19	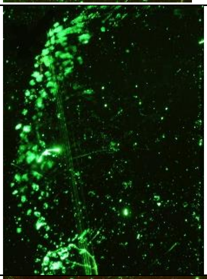	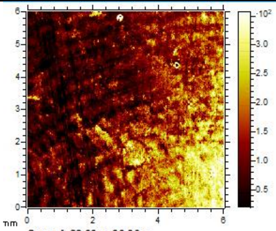	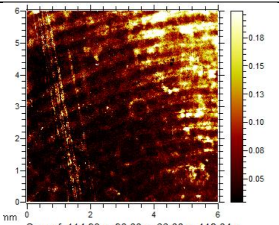
**Substrate:** Stainless-steel<br>**Condition:** Ambient<br>**Age**: 5 Months<br>**Donor**: M2<br>**Deposition:** 75	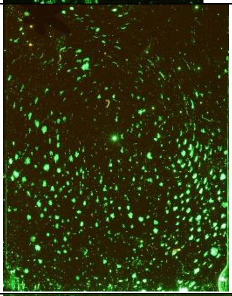	X	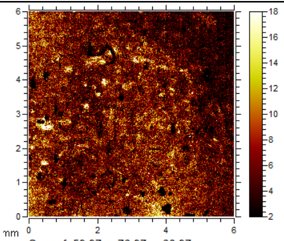
**Substrate:** Stainless-steel<br>**Condition:** Ambient<br>**Age**: 5 Months<br>**Donor**: M3<br>**Deposition:** 21	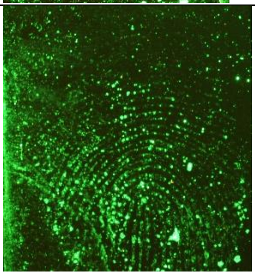	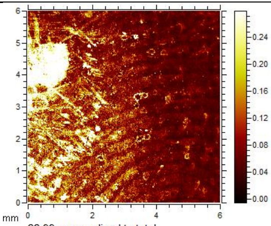	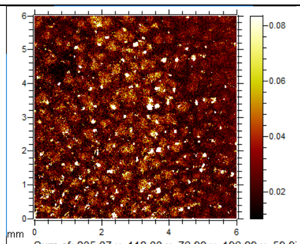
**Substrate:** Stainless-steel<br>**Condition:** Ambient<br>**Age**: 5 Months<br>**Donor**: M4<br>**Deposition:** 41	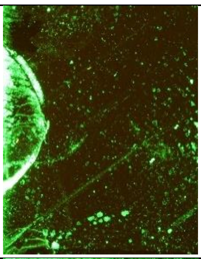	X	X
**Substrate:** Stainless-steel<br>**Condition:** Ambient<br>**Age**: 5 Months<br>**Donor**: M5<br>**Deposition:** 8	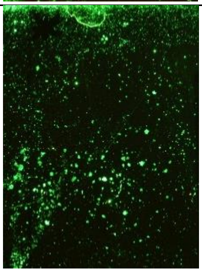	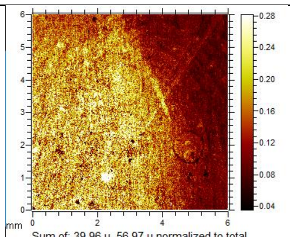	X

**Substrate:** Polyethylene **Condition:** Soil **Age**: 5 Months **Donor**: F1 **Deposition:** 1	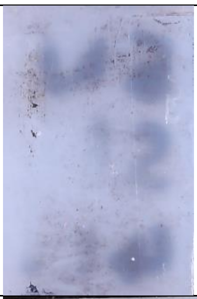	X	X
**Substrate:** Polyethylene **Condition:** Soil **Age**: 5 Months **Donor**: F2 **Deposition:** 20	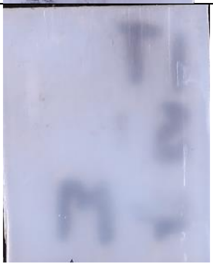	X	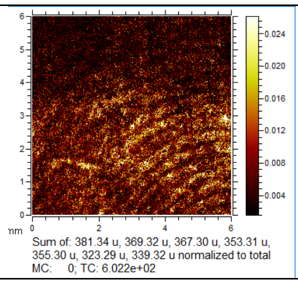
**Substrate:** Polyethylene **Condition:** Soil **Age**: 5 Months **Donor**: F3 **Deposition:** 1	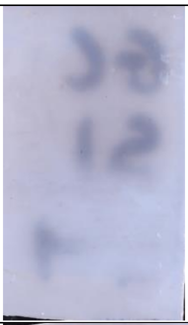	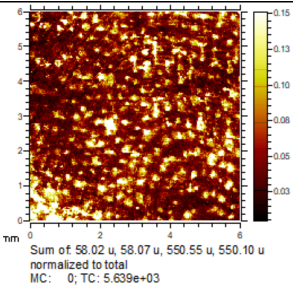	X
**Substrate:** Polyethylene **Condition:** Soil **Age**: 5 Months **Donor**: F4 **Deposition:** 1	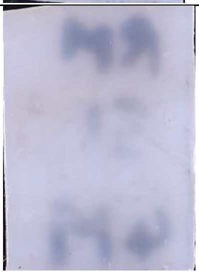	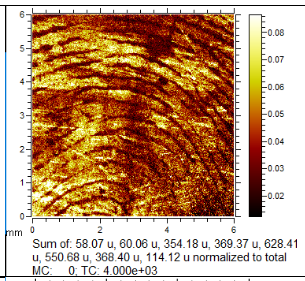	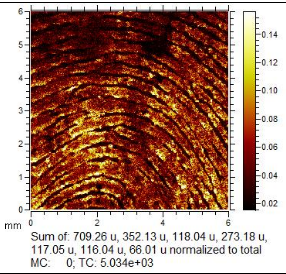
**Substrate:** Polyethylene **Condition:** Soil **Age**: 5 Months **Donor**: F5 **Deposition:** 1	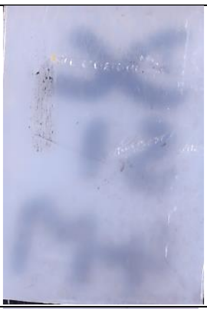	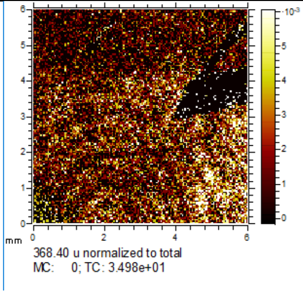	X
**Substrate:** Polyethylene **Condition:** Soil **Age**: 5 Months **Donor**: M1 **Deposition:** 1	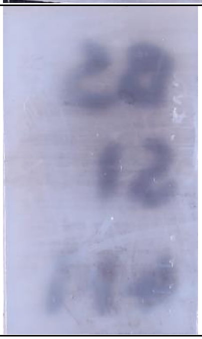	X	X

**Substrate:** Polyethylene<br>**Condition:** Soil<br>**Age**: 5 Months<br>**Donor**: M2<br>**Deposition:** 1	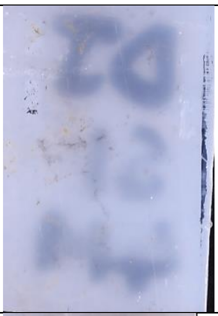	X	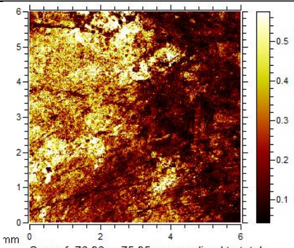<br>Sum of: 76.96 u, 75.95 u normalized to total<br>MC: 1; TC: 2.241e+04
**Substrate:** Polyethylene<br>**Condition:** Soil<br>**Age**: 5 Months<br>**Donor**: M3<br>**Deposition:** 1	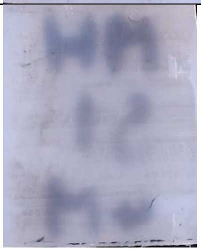	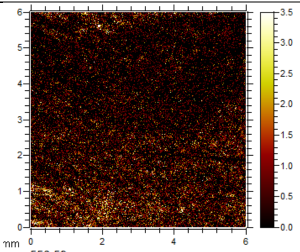<br>550.59 u<br>MC: 4; TC: 3.339e+04	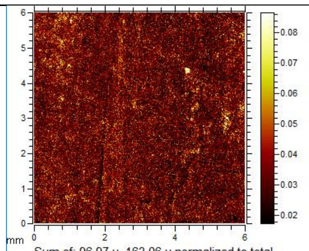<br>Sum of: 96.97 u, 163.06 u normalized to total<br>MC: 0; TC: 3.064e+03
**Substrate:** Polyethylene<br>**Condition:** Soil<br>**Age**: 5 Months<br>**Donor**: M4<br>**Deposition:** 1	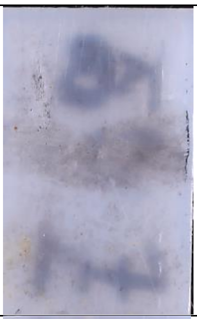	X	X
**Substrate:** Polyethylene<br>**Condition:** Soil<br>**Age**: 5 Months<br>**Donor**: M5<br>**Deposition:** 1	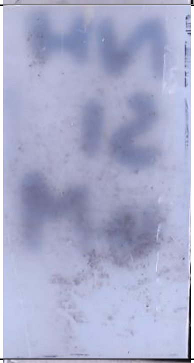	X	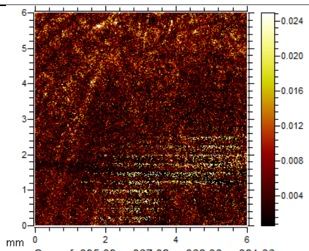<br>Sum of: 395.39 u, 367.32 u, 368.30 u, 381.36 u normalized to total<br>MC: 0; TC: 4.705e+02
**Substrate:** Stainless-steel<br>**Condition:** Soil<br>**Age**: 5 Months<br>**Donor**: F1<br>**Deposition:** 2	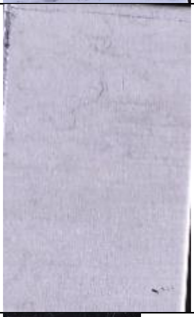	X	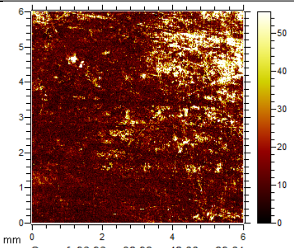<br>Sum of: 96.96 u, 32.98 u, 42.00 u, 89.01 u<br>MC: 56; TC: 1.429e+06
**Substrate:** Stainless-steel<br>**Condition:** Soil<br>**Age**: 5 Months<br>**Donor**: F2<br>**Deposition:** 1	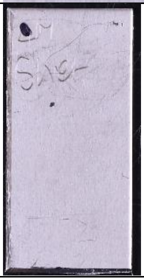	X	X

**Substrate:** Stainless-steel<br>**Condition:** Soil<br>**Age**: 5 Months<br>**Donor**: F3<br>**Deposition:** 1	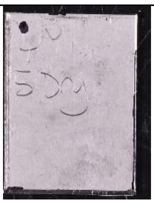	X	X
**Substrate:** Stainless-steel<br>**Condition:** Soil<br>**Age**: 5 Months<br>**Donor**: F4<br>**Deposition:** 1	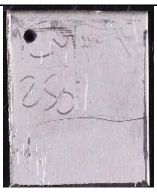	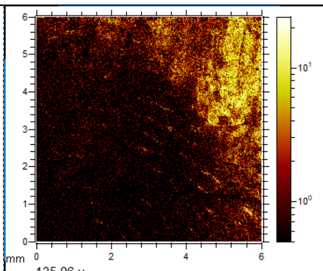	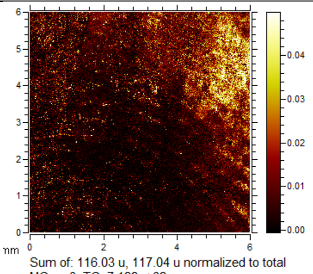
**Substrate:** Stainless-steel<br>**Condition:** Soil<br>**Age**: 5 Months<br>**Donor**: F5<br>**Deposition:** 1	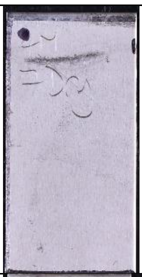	X	X
**Substrate:** Stainless-steel<br>**Condition:** Soil<br>**Age**: 5 Months<br>**Donor**: M1<br>**Deposition:** 2	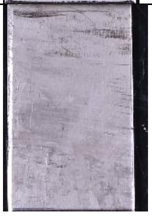	X	X
**Substrate:** Stainless-steel<br>**Condition:** Soil<br>**Age**: 5 Months<br>**Donor**: M2<br>**Deposition:** 1	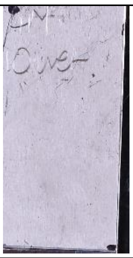	X	X
**Substrate:** Stainless-steel<br>**Condition:** Soil<br>**Age**: 5 Months<br>**Donor**: M3<br>**Deposition:** 1	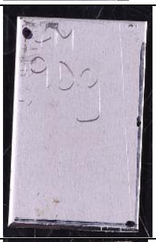	X	X
**Substrate:** Stainless-steel<br>**Condition:** Soil<br>**Age**: 5 Months<br>**Donor**: M4<br>**Deposition:** 59	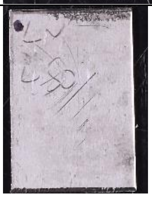	X	X

**Substrate:** Stainless-steel<br>**Condition:** Soil<br>**Age**: 5 Months<br>**Donor**: M5<br>**Deposition:** 1	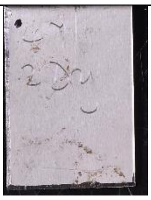	X	X
**Substrate:** Polyethylene<br>**Condition:** Water<br>**Age**: 5 Months<br>**Donor**: F1<br>**Deposition:** 6	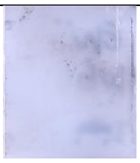	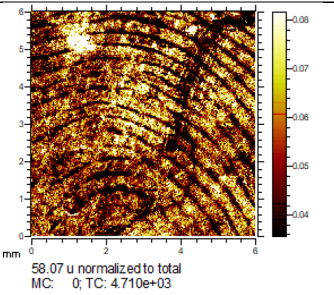<br>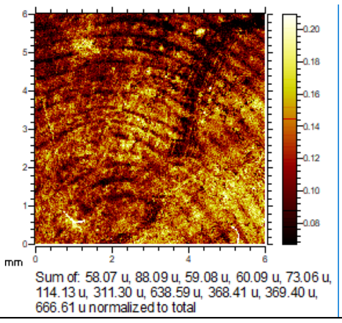	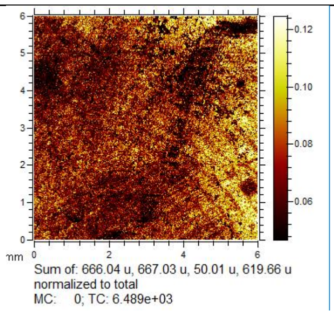
**Substrate:** Polyethylene<br>**Condition:** Water<br>**Age**: 5 Months<br>**Donor**: F2<br>**Deposition:** 1	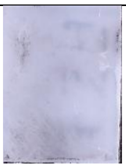	X	X
**Substrate:** Polyethylene<br>**Condition:** Water<br>**Age**: 5 Months<br>**Donor**: F3<br>**Deposition:** 1	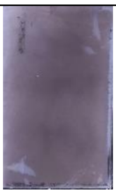	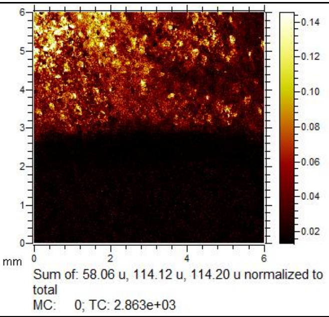	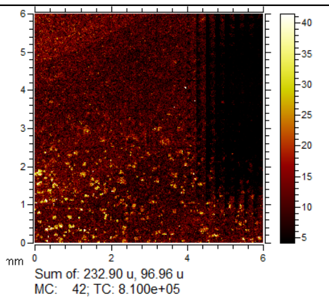
**Substrate:** Polyethylene<br>**Condition:** Water<br>**Age**: 5 Months<br>**Donor**: F4<br>**Deposition:** 1	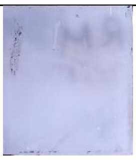	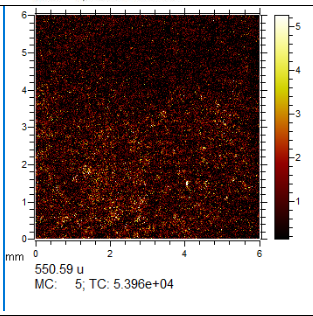	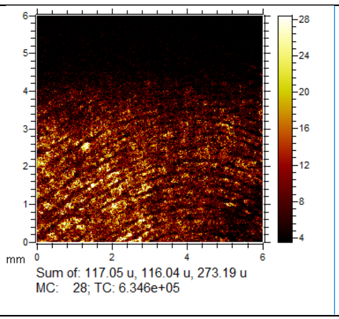

**Substrate:** Polyethylene **Condition:** Water **Age**: 5 Months **Donor**: F5 **Deposition:** 1	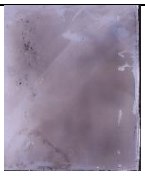	X	X
**Substrate:** Polyethylene **Condition:** Water **Age**: 5 Months **Donor**: M1 **Deposition:** 1	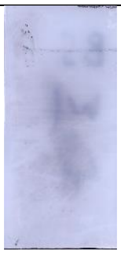	X	X
**Substrate:** Polyethylene **Condition:** Water **Age**: 5 Months **Donor**: M2 **Deposition:** 1	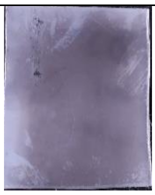	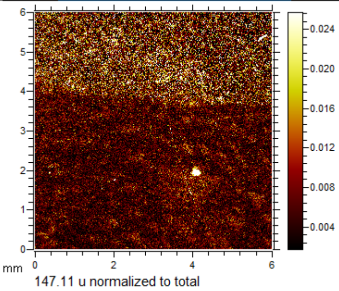	X
**Substrate:** Polyethylene **Condition:** Water **Age**: 5 Months **Donor**: M3 **Deposition:** 1	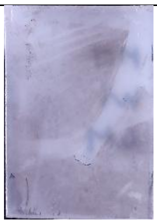	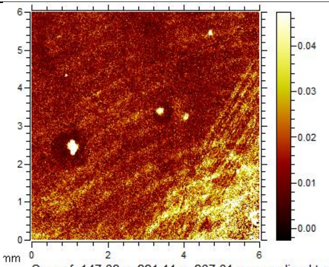 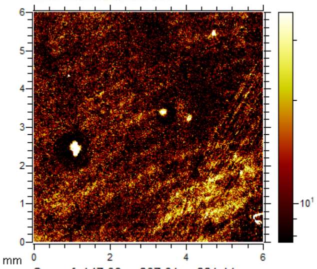	X
**Substrate:** Polyethylene **Condition:** Water **Age**: 5 Months **Donor**: M4 **Deposition:** 1	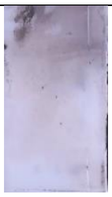	X	X

**Substrate:** Polyethylene<br>**Condition:** Water<br>**Age**: 5 Months<br>**Donor**: M5<br>**Deposition:** 1	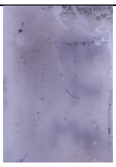	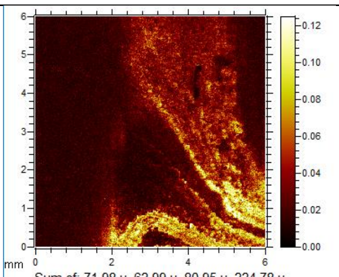	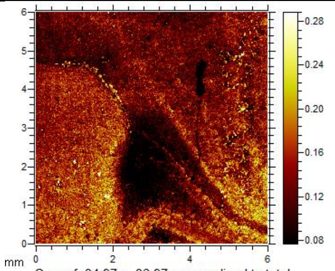
**Substrate:** Stainless-steel<br>**Condition:** Water<br>**Age**: 5 Months<br>**Donor**: F1<br>**Deposition:** 2	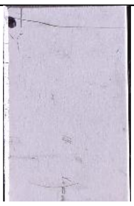	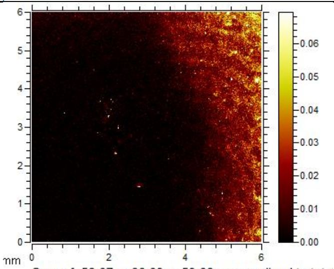	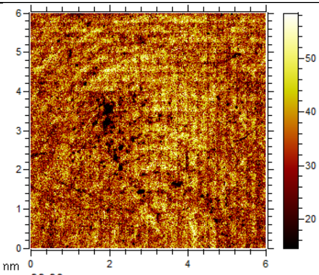
**Substrate:** Stainless-steel<br>**Condition:** Water<br>**Age**: 5 Months<br>**Donor**: F2<br>**Deposition:** 2	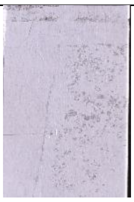	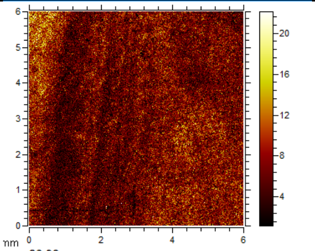<br>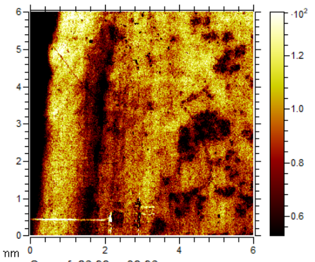	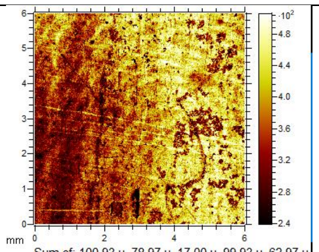<br>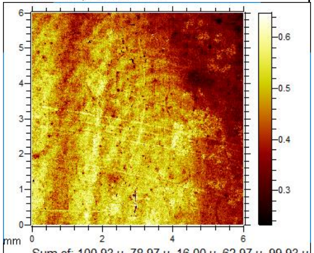
**Substrate:** Stainless-steel<br>**Condition:** Water<br>**Age**: 5 Months<br>**Donor**: F3<br>**Deposition:** 8	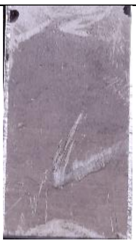	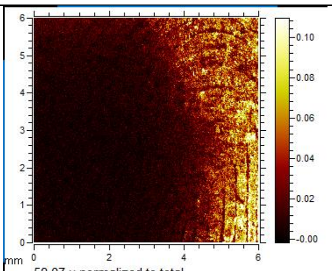	X

**Substrate:** Stainless-steel<br>**Condition:** Water<br>**Age**: 5 Months<br>**Donor**: F4<br>**Deposition:** 6	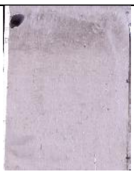	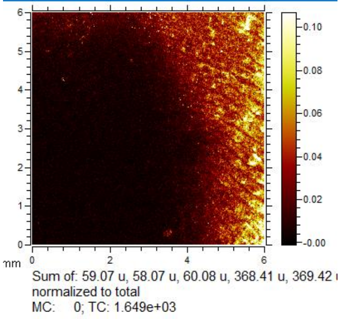	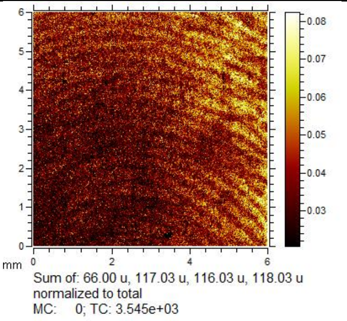
**Substrate:** Stainless-steel<br>**Condition:** Water<br>**Age**: 5 Months<br>**Donor**: F5<br>**Deposition:** 1	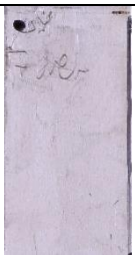	X	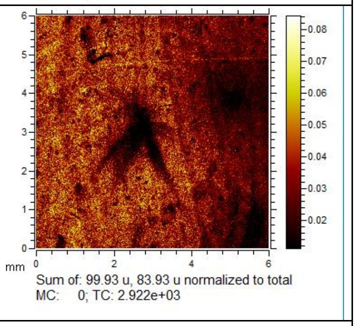
**Substrate:** Stainless-steel<br>**Condition:** Water<br>**Age**: 5 Months<br>**Donor**: M1<br>**Deposition:** 9	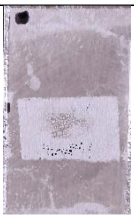	X	X
**Substrate:** Stainless-steel<br>**Condition:** Water<br>**Age**: 5 Months<br>**Donor**: M2<br>**Deposition:** 1	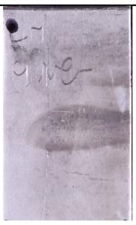	X	X
**Substrate:** Stainless-steel<br>**Condition:** Water<br>**Age**: 5 Months<br>**Donor**: M3<br>**Deposition:** 8	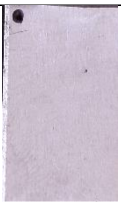	X	X
**Substrate:** Stainless-steel<br>**Condition:** Water<br>**Age**: 5 Months<br>**Donor**: M4<br>**Deposition:** 2	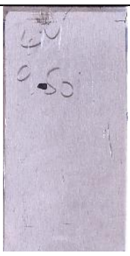	X	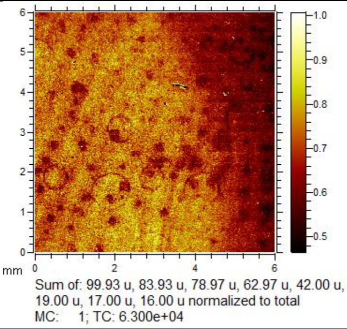

**Substrate:** Stainless-steel<br>**Condition:** Water<br>**Age**: 5 Months<br>**Donor**: M5<br>**Deposition:** 2	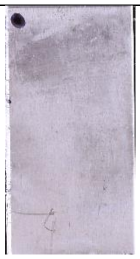	X	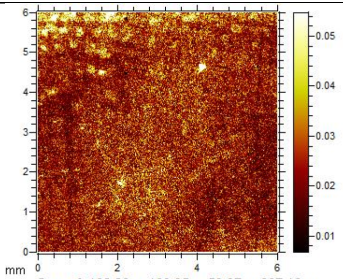

Supporting Information Table S2 – Table of common m/z values yielded from positive mode ToF-SIMS for male and female donors on polyethylene and stainless-steel after aging for 1 month or 5 months under all conditions (ambient, soil or water). The number indicates in how many donors the m/z value yielded an image of ridge detail, for each condition.

	1 Month											5 Months								
m/z	Polyethylene Ambient Female	Polyethylene Ambient Male	Polyethylene Water Female	Polyethylene Water Male	Polyethylene Soil Female	Polyethylene Soil Male	Stainless-steel Ambient Female	Stainless-steel Ambient Male	Stainless-steel Water Male	Stainless-steel Soil Female	Stainless-steel Soil Male	Polyethylene Ambient Female	Polyethylene Ambient Male	Polyethylene Water Female	Polyethylene Water Male	Polyethylene Soil Female	Polyethylene Soil Male	Stainless-steel Ambient Male	Stainless-steel Water Female	Total
23	1	2				2		1			1	1						2		10
26		2	1					1	1											5
27		1	1	1	1	1		1	1										1	8
28		2	1	1		2		2	1		1									10
29		3	1	1	1	1		1												8
30			1	2	3	3		1	1		1									12
39	1	3	2	1	1	1	1	1				1						1	1	14
40	1					1		2	2									1		7
41	1		1	1	1	2														6
42			1	1	2	1		1		1	1									8
43	1	3	1	1	1			1			1									9
44		2	1	2	3	1		1	1	1	1									13
45	2	3				1				1										7
52	2	3	1					1												7
55		3	1	1	1			1												7
56			2		1	1		2	1	1	2									10
57	1	2	1	1		1	1	2	2		2							1		14
58	2		3	3	4	2		1		1	1			2		2			3	24
59	1		2		4	1		1		1	1	1	2	1					2	17
60			3	1	4						1			1		1			2	13

70		1	1		1	1		1	1	1	1									8
72			1		2	1		1		1	1				1					8
73		1	1			1		1						1						5
80	1	3			1			1				1								7
82		1	1					1	1		1									5
84			1		2	1				1	1									6
86			1		3					1	1									6
88			2	1	3	1		1						1						9
98	4	5	1		1	2		1		1		2	3							20
99	1	2	1	1	2	1														8
100	1		1		3															5
102	1	1	1		1	1		1												6
114	1	1	2	1	4	1		1						2		1				14
118	1	2				1		1												5
119		1		1		1		1		1										5
124	1	2	1					1				2	2							9
126	2	4	1		2	1		1				1	3							15
127		3				1		1					1							6
128		1	2		4	1														8
132	1	1				1		1					1							5
140	2	2	1		1	1		1					3							11
142	1	1	2		4	1		1												10
145	1	2			1	1		1												6
147		1	1					1							2					5
154	4	5	1		1			1				2	4							18
156	1	2	2		3	1		1												10
170	1		3		4	1		1												10
171	2	3						1												6
184			1		3	1														5
198			2		4	1														7
212			2	1	4	1														8
226			1		4															5
268			1	1	4	1														7
283			1	1	2	1		1												6
290	3	3						2					1							9
291	1	3						2												6
296			2	1	4	1														8
297			1	1	3	1														6
298			1		2	1				1										5
304	2	2				1		1												6
311			2	1	2	1								1						7
318	3	3						2				1	1							10
319	1	3						2					1							7
324			1		3	1														5
332	3	3				1		2												9
333	1	2				1		1												5

334	3	3						2												8
338			1		4															5
340			3		3															6
346	1	3						1												5
348	1	3						1												5
352			2		4	1														7
354			1		3											1				5
360	3	3		1		1		2												10
361	1	2				1	1	1				1								7
362	5	4						2				1								12
363	4	3						2												9
364	3	3	1		1			1												9
366			2		4	1														7
368			3		3									1		2			1	10
369			3		3									1		1			1	9
378	1	2	1		1			1												6
380			1		4	1		1												7
408		1	1		1	1		1												5
495			1	1	3															5
523			1	1	3															5
524			1	2	3															6
550			1	1	2											1				5
551			2	1	2	1								1		2	1			10

Supporting Information Table S3 – Table of common m/z values yielded from negative mode ToF-SIMS for male and female donors on polyethylene and stainless-steel after aging for 1 month or 5 months under all conditions (ambient, soil or water). The number indicates in how many donors the m/z value yielded an image of ridge detail, for each condition.

	1 month											5 months								
m/z	Polyethylene Ambient Female	Polyethylene Ambient Male	Polyethylene Water Female	Polyethylene Water Male	Polyethylene Soil Female	Polyethylene Soil Male	Stainless-steel Ambient Female	Stainless-steel Ambient Male	Stainless-steel Water Male	Stainless-steel Soil Female	Stainless-steel Soil Male	Female Polyethylene Female	Polyethylene Ambient Male	Polyethylene Water Female	Polyethylene Water Male	Polyethylene Soil Female	Polyethylene Soil Male	Stainless-steel Ambient Female	Stainless-steel Ambient Male	Total
16						2		1	1				1							5
17				1		2		1										1		5
26	5	5	1	1	2	3		1	1	2	1	3	4			1			1	31
27	2	4	1							1	1	2	4						1	16
35	1				1	1		1	1		1		1		1					8

40	1	4	1	1	2	1					1	2	3							16
41	1				2	1												1	1	6
42	1	1		1	2	2			1	1	1	1	3					1	1	16
50	4	4	1		1	1			1			3	4	1						20
52	5	5	1					2				4	4					1	2	24
53		2						1				1	2							6
60						3	1			1									2	7
61						2	1			1									2	6
62		4			1	1		2				1	1							10
63				1		1		1	1									1		5
64	1	3	1		1	1		1				2	2						1	13
65		1	1		1	1							1							5
66	5	5	2	2	2	2		3	1			4	4			1			1	32
67	5	5	1					3				4	4						1	23
68	2	4	1		1			2				2	4						1	17
74	1	4	1					1				2	3							12
76	1	4	1			1		1				2	4				1			15
77		1	1			2	1			1			1				1		2	10
78	1	4	1					1				2	4							13
79				1		1		1	1	1								1		6
80	1			1	2	1	1	1					1	1						9
84			1		1								1							3
88	4	3	1			1		1				2	4						1	17
89		2						1				2	3						1	9
90	5	5	1			1		2				4	4						1	23
91	1	3	1					2				2	3						1	13
92	1	3	1									2	3							10
96	4	5	1		2							4	3							19
97		2	1	1	2	2						1	1	1			1			12
98	1	3	1		2							1	2							10
100		1	1					1				1	1							5
101			1			1		2												4
102		1	1					1				1	1							5
103	2	5	1			1		1				4	4							18
104	1	4	1					1				2	2							11
105	1	2	1					1				2	3							10
106	1	3	1					1				2	4							12
110			1		2			0				1	1							5
112	5	5	1					2				4	4					1	2	24
113	3	3	1					2				2	4						1	16
114	1	4	1					1				2	4						1	14
115	3	4	1	1		2		2	1		1	2	4						1	22
116	1	3	2		1	1		1				2	2	1		1			1	16
117		1	1			2		1			1	2	2	1		1			1	13
118	4	5	1					1				3	4			1			1	20
119	1	2	1			2						2	2							10

124		1	1		2	1							1							6
125	1	1	1					1				1	3							8
126	2	4	1	1				2				3	4							17
127		1	1					1					2							5
128		2	1					1				2	3							9
130	1	2	1					2				1	3							10
131		3	1					1	1		1	2	3							12
136	2	3	1			1		1				2	3							13
137	1	3	1			1		1				2	3							12
138	1	2	1		1			1				2	3							11
139		3	1		1	1		1				2	3							12
140	4	4	1					2				3	4						1	19
141		2	1					1					2							6
142		2	1	1				1				2	4						1	12
143	1	2	1	1				2				1	2							10
144		1	1									1	2							5
146		2	1									2	4							9
150	1	4	1					1			1	2	4				1			15
151	1	3						1				2	3							10
152		3	1									2	3							9
153		1	1					1				1	1							5
154		2	1					1				2	1							7
161	1	4	1					1				2	3			1				13
162		2	1					1				2	3							9
163	3	5						2				3	4						1	18
164	1	4	1					1				3	4							14
165		1	1					1				1	1							5
166	1	2	1					1				2	3							10
167		1	1		1			1				2	1							7
168	1	2	1					1				2	2							9
170		1	1					1				1	1							5
175		3	1					1				2	2							9
176	1	3	1					1				2	4							12
177	5	5	1					2				4	4							21
178	4	4	1					2				3	4							18
179		1	1			2		1				1	2							8
180		3	1					1				1	3							9
183	1		1		1		1	1						1						6
187		1	1					1				1	1							5
188		2	1					1				2	1							7
191		3	1					1				2	3							10
192		1	1					1				1	2							6
193		1	1					1				2	4							9
207			1									2	4							7
208		1	1					1				2	1							6
210		2	1	1	1			1				2	3						1	12

214	3	4	1					1				3	4							16
215		2	1					1				2	2							8
224	4	5	1					2				3	4					1	1	21
225		2	1					1				2	1							7
226	1	1	1					1				1	1							6
228	2	3	1					1				2	4							13
235	5	5	1					2				4	4						2	23
236	5	4	1					2				4	4							20
237	1	3	1					2				3	4							14
238		1	1					1				1	1							5
249	1	4	1					1				2	4							13
250		1	1					1				1	1							5
251		1	1					1				1	2							6
254			1									2	4							7
261	1	3	1					1				2	3						1	12
265			1	1	1							1	3							7
266			1		1							2	4							8
273			2		1									1		1				5
275		3	1					1				2	3							10
286		2	1					1				2	3							9
287		1	1					1				1	1							5
288		3	1					1				2	3							10
302		3	1					1				2	4							11
311	1	1	1					1												4
333			1					1				2	1							5
334		1	1					1				2	1							6
339	1		1		2			1								1				6
349		3	1					1				2	2							9
361		3	1					1				2	3							10
362		3	1					1				2	2							9
666	2	1	1					1					1	1						7
667	2	1	1					2					1	1						8
668	1	1	1					1					1	1						6
